# Molecular assembly as a universal biosignature measurable by mass spectrometry

**DOI:** 10.1073/pnas.2619198123

**Published:** 2026-08-10

**Authors:** Lindsay A. Rutter, Abhishek Sharma, Ian Seet, David Obeh Alobo, An Goto, Leroy Cronin

**Affiliations:** ^a^ School of Chemistry, University of Glasgow, Glasgow G12 8QQ, United Kingdom

**Keywords:** assembly theory, astrobiology, complexity, machine learning

## Abstract

Detecting life beyond Earth requires biosignatures that do not depend on the chemistry of known organisms. Molecular assembly (MA), derived from Assembly Theory, quantifies how difficult it is to build a molecule from basic building blocks, linking complexity directly to selection and evolution. Here, we show that MA can serve as a universal biosignature that is both interpretable and experimentally measurable. Unlike information-theoretic measures, MA can be inferred directly from mass spectrometry data without structural elucidation. We demonstrate this using a machine learning model trained on standardized single-stage (MS^1^) spectra, which predicts MA with three-fold lower error than baseline methods. Simulated multistage (MS^n^) data reveal that small instrumental variations can double prediction error, highlighting the importance of calibration. These findings establish molecular assembly as a physically grounded, quantifiable biosignature measurable by mass spectrometry whose interpretation depends on careful control of instrumental effects, offering a scalable route to life detection on future planetary missions.

## Significance

This work introduces molecular assembly (MA) as a universal, quantitative biosignature that measures how difficult it is to build a molecule. By linking chemical complexity to selection and evolution, MA can identify life independent of specific biochemistry. Using machine learning to infer MA from mass spectrometry data, it is possible to detect signatures of directed chemical organization, the defining property of living systems, without knowing molecular structures in advance. This framework converts raw spectral data into a measurable indicator of life’s causal imprint, offering a practical, theoretically grounded route for agnostic life detection on proposed planetary missions. The reliability of MA-based predictions can be further enhanced by careful calibration and standardized instrument settings for mass spectrometers for planetary missions.

Despite advances in our exploration of the Universe, we have yet to determine whether extraterrestrial life exists. At the same time, even defining life itself remains one of the most enigmatic challenges in science (1). Our only known example is terrestrial life, thought to have evolved from a common ancestor under conditions specific to Earth (2–4). Traditionally, life detection missions have targeted highly specific biosignatures (chemical or physical indicators of life), while limiting the search space by relying on assumptions about life as we know it. The Viking’s Labeled Release experiment remains a seminal milestone in astrobiology, proving the first in situ metabolic assay of Martian surface material (5). A critical issue is that while a radioactive gas, the GCMS did not detect the organics expected, but one argument made that the concentration of microbes was too low for GCMS, but high enough for the labeled release experiment. The absence of a clear definition of life, coupled with the difficulty of interpreting the alien environments in which it might arise, has led to inconclusive outcomes.

Two notable examples of atmospheric gases within our Solar System are methane (CH_4_) in the Martian atmosphere and phosphine (PH_3_) in the Venusian atmosphere (6, 7), which are often discussed in the context of possible biosignatures. While it is clear that methane can be formed biologically via methanogens (microorganisms that generate methane) (8), the biological processes thought to be involved in direct phosphine production are less clear (9). However, these gases are also chemically simple and hence, possess a higher chance of forming through abiotic (nonbiological) processes, such as volcanic eruptions and geothermal activity (10, 11). This interpretation is further complicated by the fact that both molecules are reactive and can be unstable in planetary atmospheres, undergoing decomposition through ultraviolet photolysis, atmospheric chemistry, and reactions with mineral surfaces (12–15). Scientists have speculated about biological origins after detecting them at levels that purportedly exceed what known environmental processes alone can easily sustain (6, 16). Yet the dynamic balance between production and destruction of these gases makes such speculation difficult to confirm (17). Similar ambiguities apply to biosignature candidates detected beyond our Solar System. One prominent example is dimethyl sulfide (DMS, C_2_H_6_S), tentatively identified in the atmosphere of the exoplanet K2-18b, located over 100 light-years away (18). K2-18b is classified as a Hycean planet, a type of temperate world thought to host a subsurface ocean beneath a hydrogen-rich atmosphere (19). Recently, it has been shown that the presence of red noise negatively impacts mid-IR data in the Mid-Infrared Instrument (MIRI) transit spectrum, and the evidence is not conclusive (20). On Earth, DMS is linked to marine microbial activity and decomposition, which is why it has been suggested as a potential biosignature candidate (21). However, it has also been identified in abiotic environments, such as cometary dust (22), and can be synthesized under prebiotic laboratory conditions (23). Disentangling whether these candidate molecules originate from biological or nonbiological sources requires quantifying the full range of environmental processes that could produce or destroy them, and the possibility that unfamiliar forms of life (potentially radically different from those on Earth) might be responsible for their presence. Given these many uncertainties, it remains difficult to assert that the presence of simple molecules (whether methane, phosphine, or DMS) provides clear evidence of alien life (24, 25).

**Fig. 1.**
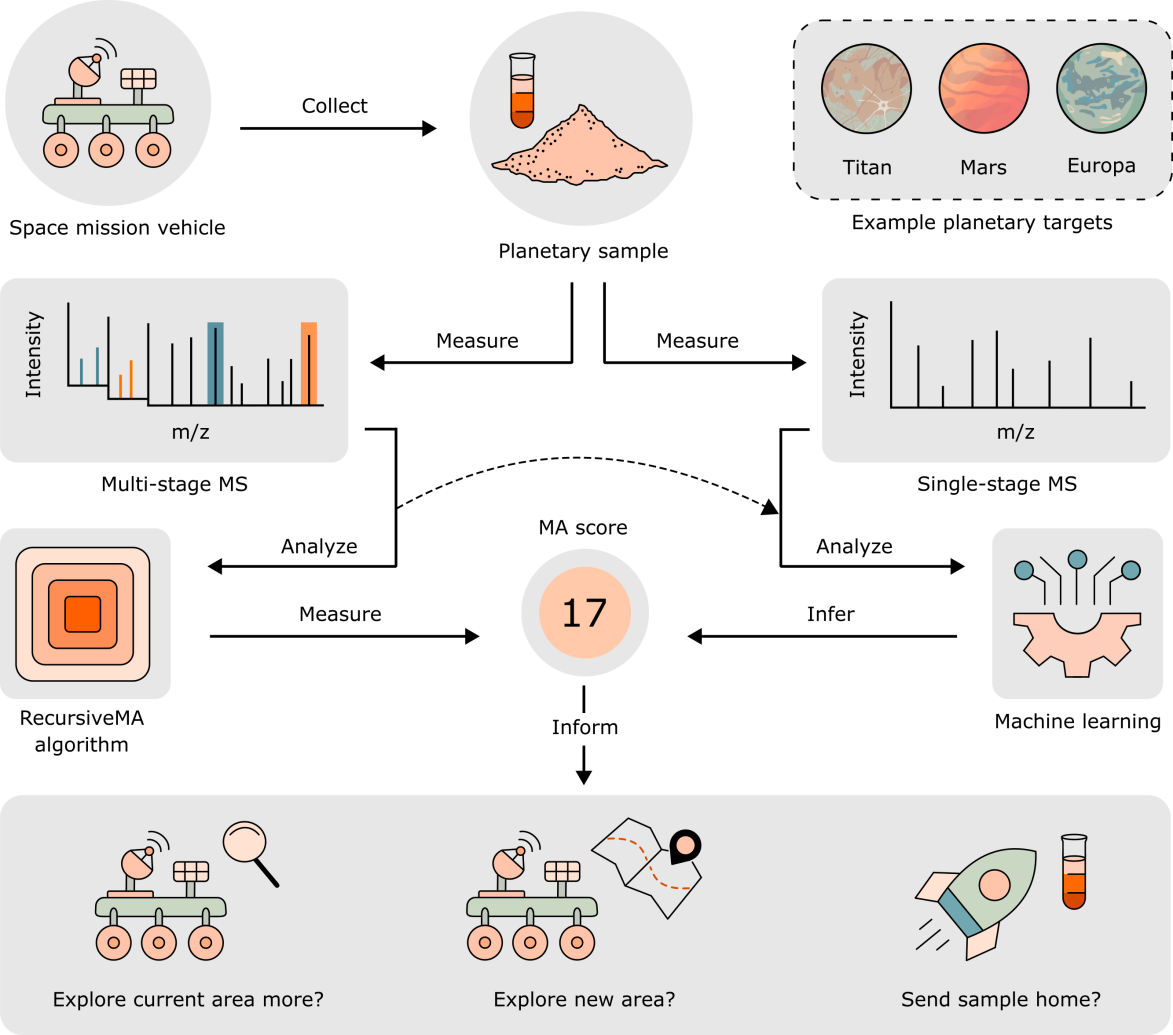
Schematic overview of deploying MA as an agnostic biosignature. Proposed life detection missions in the Solar System can collect samples in situ and predict MA scores directly from MS data without the need for structural elucidation, which may be difficult for unknown molecules in extraterrestrial environments. Space missions that use MS^n^ can estimate MA scores with the RecursiveMA algorithm (30). In the current paper, we show that space missions that use MS^1^ (and optionally MS^n^) can infer MA scores with ML.

There is a growing interest in “agnostic” life detection strategies: methods that can identify molecular signs of life without needing an exact understanding of what that life might look like, or how it might interact with a complex, poorly characterized environment (26–28). One alternative approach for distinguishing between abiotic and biotic molecules is to estimate their assembly indices; previous studies have shown an abiotic-to-biotic threshold at an assembly index of ca. 15 (29). The measured assembly index, defined as the number of steps along the shortest construction pathway, is fixed for each molecule and does not depend on external conditions, such as the environment in which the molecule might be detected or the specific ‘alien’ biochemistry from which it might arise. This makes MA a more fundamental property of a molecule beyond classic complexity scores. As MA is independent of such difficult-to-quantify environmental factors, MA could be considered an agnostic biosignatures. Inspired by the previous studies, herein, depending on the observed experimental dataset, we propose an approach to quantify life detection by combining the fundamentals of AT with machine learning algorithms, Fig. 1. Our approach considers the ability of AT to detect and quantify degree of selection and evolution, intrinsic signatures of life, at the molecular level.

## Molecular Complexity Scores as Potential Biosignatures and Their Measurement.

**Fig. 2.**
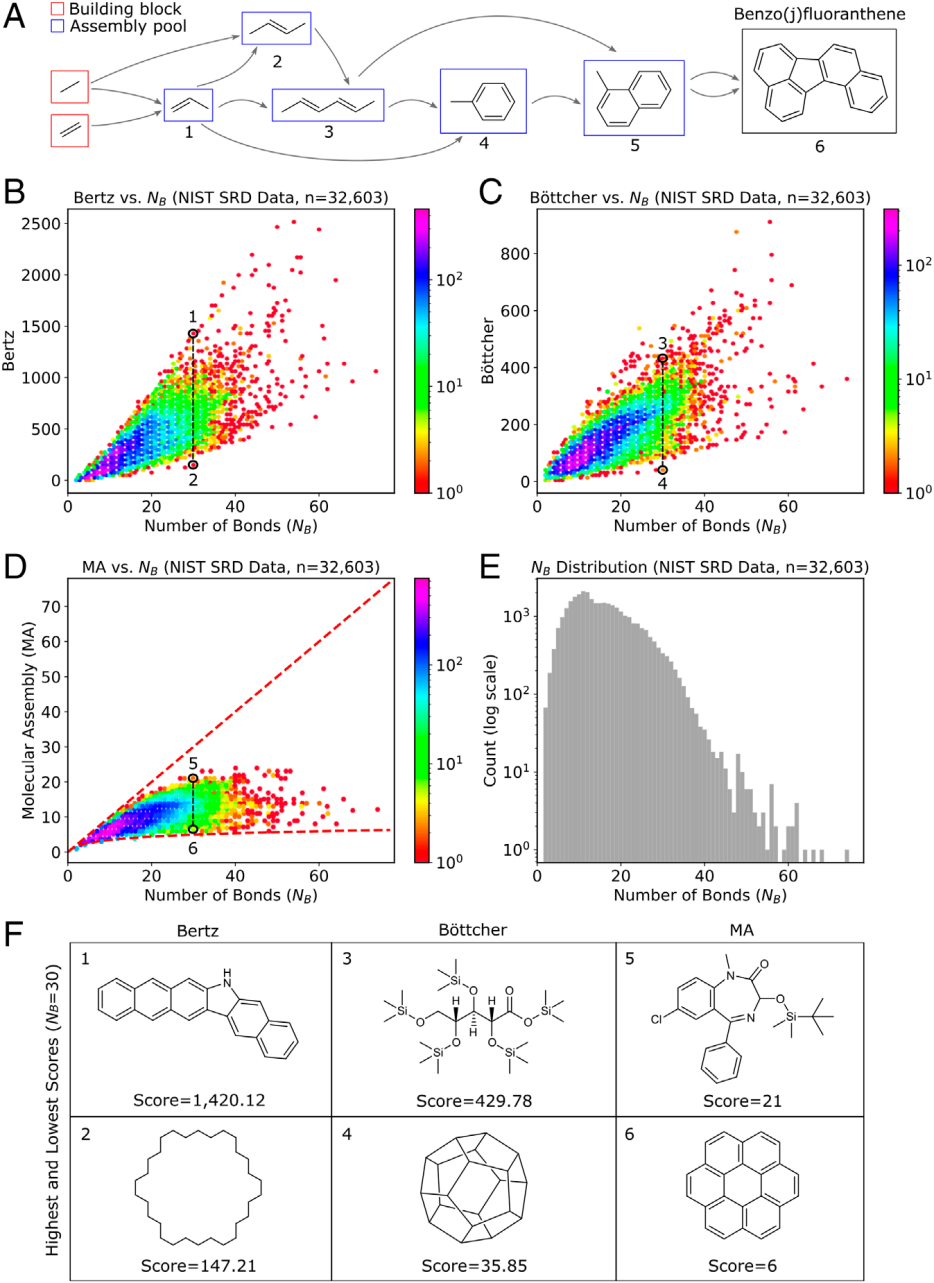
Relationship between complexity scores and *N_B_*. (*A*) Example shortest pathway construction for a molecule with MA = 6. (*B*–*D*) Scatterplots of complexity scores vs. *N_B_*. The two dotted red lines demonstrate mathematically derivable bounds of MA scores, with an upper bound of $\mathrm{MA}=N_{B}-1$ and a lower bound of $\mathrm{MA}={\text{log}}_{2}\left(N_{B}\right)$. The color legends indicate the count of molecules on a logarithm scale. (*E*) The distribution of *N_B_* for the 32,603 molecules with MS^1^ on the NIST SRD database (35). (*F*) Highest and lowest scoring molecules with $N_{B}=30$.

Several molecular complexity methods have been proposed, such as those by Bertz and Böttcher, which aim to quantify the structural or informational richness of a molecule (31, 32). Considering graph theoretic and information theoretic features, it estimates molecular complexity by treating the number of graph invariants of a specified type and distinct heteroatoms in a molecular graph as discrete states in Shannon’s formula for information entropy. The Böttcher score, another well-established molecular complexity metric, estimates the information content in each atom’s microenvironment. It does so by assessing stereogenicity, the diversity of heavy atom and bond types, and chemical equivalence (32). Both scores use information-theoretic approaches to quantify the structural diversity of a molecule, independent of how it was built. More recently, a new approach has been developed from a theoretical framework known as Assembly Theory (AT), which seeks to explain how complex objects arise through processes of selection and evolution (33). To quantify the degree of selection required to construct an ensemble of molecules, AT utilizes the assembly index and the copy number of the observed objects, where the amount of selection is quantified by the assembly of the ensemble (*A*):

[1] $$A=\sum_{i\epsilon n_{i}>1}^{N}e^{a_{i}}\left(\frac{n_{i}}{N_{T}}\right),$$

where *a_i_* is the assembly index of the *i^th^* object, *n_i_* is its copy number of the *i^th^* object, *N* is the total number of unique objects, *e* is Euler’s number, and *N_T_* is the total number of objects in the ensemble. Here, the object’s assembly index is defined as the length of its shortest construction pathway, that is, the minimum number of discrete steps required to build it from basic building blocks and objects in the assembly pool (i.e., intermediate objects produced in earlier steps that can be reused in subsequent ones) (Fig. 2*A*) (33). When the object is a molecule, its assembly index is termed molecular assembly (MA). Because MA depends only on the shortest path, and this can be measured experimentally, this approach to molecular complexity provides a more objective measure, which is less susceptible to false positives, Fig. 2*A* (33). Both features in Eq. **1**: assembly index and copy number are, in principle, experimentally measurable. The central focus of the study is the measurement of assembly index from single-stage or multistage mass spectrometry. In principle, the MS intensity can be used to infer the copy number or concentration of the molecule in the sample subject to careful calibration and modeling. When working with a mixture of molecules, their assembly score is termed their joint molecular assembly (JMA), which represents the shortest pathway to construct all molecules in the ensemble (33, 34). Whereas Bertz and Böttcher quantify molecular properties using information-theoretic methods without regard to construction history, MA uses algorithmic approaches to compute the shortest construction pathway, capturing history-dependent complexity.

AT’s core premise is that a molecule with a high MA is statistically unlikely to form, especially in large quantities (i.e., high copy number), through random abiotic processes. On a barren world, the exploration of molecular space would likely be undirected, favoring the spontaneous formation of simpler molecules of lower complexity (i.e., molecules with low MA), leading to a combinatorial explosion. However, under selective pressures, this exploration becomes more directed, favoring the emergence of increasingly complex molecules with longer construction pathways and greater shared historical contingencies (i.e., molecules that share more assembly pool objects with one another). The most plausible source of such pressures is biology, which evolved to encode information that constrains chemical reaction networks, guiding the reliable synthesis of highly specific and complex molecules. Consistent with this idea, experimental findings suggest that molecules with MA scores above specific thresholds may serve as reliable indicators of biology (29).

Many proposed mission concepts currently in development—including the Europa Lander, the Enceladus Orbilander, and the currently planned Dragonfly expedition to Titan—will potentially carry mass spectrometers to analyze molecular samples directly on planetary surfaces (36). In these remote environments, where structural elucidation is infeasible due to unfamiliar molecules or limited instrument resolution, the ability to measure molecular complexity directly from mass spectrometry (MS) data will be critical. Additional complexity arises from the combination of other factors such as effects of sample preparation, analyte extraction or purification, reactive mineral matrices, and the availability of appropriate standards. The detections of chlorinated, sulfur-bearing, aromatic, and aliphatic organic compounds by the Sample Analysis at Mars (SAM) instrumentation suite on NASA’s Curiosity rover in Gale crater sediments rely on extremely cautious laboratory benchmarking and interpretation (37–40). Among existing approaches to molecular complexity, MA can be measured by a range of techniques, including tandem mass spectrometry (MS^n^) (30). In MS^n^, a parent molecule is sequentially fragmented (i.e., fragmentation of fragments) into smaller ions through collision-induced dissociation, and this process is repeated across multiple stages until the smallest fragments can no longer be detected. The resulting fragmentation tree is created by searching combinations of masses of fragments. The first-order MA can be estimated from the parent mass, but it is prone to deviating from the actual MA due to the presence of heavy atoms and repeated structures. These features can be observed in fragmentation trees, which can be used to improve the estimation of the MA. To operationalize this approach, the RecursiveMA algorithm (30) was developed to successfully quantify MA scores directly from high-resolution, MS^n^ data (Fig. 1). In contrast, scores like Bertz and Böttcher lack this physical connection to experimental data, as they rely on global, information-theoretic properties that do not reflect stepwise construction or fragmentation, i.e., there is no physical technique that can be used to measure the complexity of the sample directly unless the structure has already been determined.

An effective agnostic biosignature must satisfy two key criteria: it must be interpretable, and it must be experimentally determinable. As previously outlined, MA satisfies both. First, it is interpretable: it exhibits threshold scores above which molecules are unlikely to arise abiotically, as they suggest a history of directed construction by life (29). MA’s interpretability comes from its capacity to quantify rigorously how likely or unlikely it is to observe a molecule or an ensemble of molecules over the exploding combinatorial chemical spaces. Additionally, this approach does not include any observational biases by considering the shortest construction pathway, which makes it an intrinsic property of the molecule. Second, it can be measured: this is done by applying RecursiveMA to MS^n^ data (30). Importantly, this does not require structural elucidation, making it highly suitable for unknown compounds in extraterrestrial settings. For these reasons, MA has been specifically proposed as a tool for agnostic life detection (29). These are two key features of MA that make it a useful candidate to be explored by machine learning methods.

Considering this, an ideal strategy would be to deduce MA directly from MS^n^ data using RecursiveMA. However, RecursiveMA cannot be used to measure MA from single-stage mass spectrometry (MS^1^) data directly, because it requires fragment information across multiple stages (30). It is important to note that EI produces fragmentation during ionization and can yield extensive fragment-ion patterns, sometimes with little or no intact molecular ion, whereas ESI more often preserves a precursor ion for subsequent MS/MS or MS^n^ analysis. Although EI spectra can resemble the information content of MS/MS, they are not formally equivalent because tandem MS involves the deliberate isolation of a precursor ion followed by controlled fragmentation. Even when MS^n^ data are available, limited fragmentation stages, poor fragmentation behavior, or limited mass resolution may prevent accurate MA determination. In such cases, it is worth exploring whether machine learning (ML) could assist in predicting MA scores from available MS data (Fig. 1) (41). To investigate this possibility, we evaluated the prediction of MA from MS^1^ data acquired under conditions like those proposed for missions to Titan and Europa. These missions will use gas chromatography-mass spectrometry (GC–MS) with electron ionization (EI), which produces MS^1^ data. In this study, we trained ML models on data from the NIST (National Institute of Standards and Technology) Chemistry WebBook [Standard Reference Database (SRD) 69] (35), a curated repository of EI GC–MS^1^ data. We found that XGBoost (42), a gradient-boosted decision tree model, substantially outperforms baseline models (41). The model also generalizes well to other MS^1^ databases. Using simulated MS^n^ data, we further show that model accuracy depends on consistent instrument parameters (such as ionization energy), highlighting the importance of using standardized data. Taken together, these findings suggest that when algorithmic approaches are not feasible, ML models trained on relevant, standardized MS data offer a viable alternative for in situ estimation of molecular assembly. This approach can help prioritize, in real time, which regions should be further explored, and which samples should be cached for future return to Earth.

## Rationale for Selecting MA Index over Conventional Molecular Complexity Scores.

In this section, we use theoretical reasoning and large-scale analysis to identify MA as the most suitable candidate for agnostic biosignature detection, based on its interpretability. We first compare how three candidate approaches (Bertz, Böttcher, and MA) scale with the number of bonds (*N_B_*) by plotting this information for the set of molecules used in our ML model (i.e., for all molecules with MS^1^ data in the NIST SRD database) (Fig. 2 *B*–*D*). We found that Bertz increases more than linearly with *N_B_* (superlinear growth), Böttcher increases approximately linearly, and MA increases less than linearly (sublinear growth), and these findings were consistent when we examined databases larger than NIST SRD, Table 1.

**Table 1.** Relationship between complexity scores and molecular size

β (R^2^)	NIST SRD (n = 32,603)	COCONUT 2.0 (n = 526,968)	PubChem (n = 99,129,693)
Bertz	1.46 (0.76)	1.34 (0.79)	1.49 (0.85)
Böttcher	0.96 (0.67)	1.09 (0.74)	1.00 (0.74)
MA	0.74 (0.74)	0.68 (0.64)	0.74 (0.72)

Estimated power-law scaling between complexity scores (MA, Böttcher, and Bertz) and number of bonds ($N_{B}$), based on log–log regression. The scaling exponent (β) indicates whether each measure grows superlinearly (β > 1), linearly (β ≈ 1), or sublinearly (β < 1) with $N_{B}$. R^2^ values indicate the goodness-of-fit for the log–log linear model. The results were consistent between three databases (NIST SRD, COCONUT 2.0, and PubChem).

Bertz and Böttcher have variances that increase with increasing $N_{B}$, as evidenced by their funnel-shaped distributions in Fig. 2 *B* and *C*. The theoretical bounds of MA scores also exhibit this feature, as shown by the two dotted red lines in Fig. 2*D*. However, the observed molecules do not exhibit such variances that increases with $N_{B}$ in their MA: with increasing $N_{B}$, we no longer find any molecules that adhere to the upper bound of $\mathrm{MA}=N_{B}-1$, because large molecules form in high copy numbers more readily if they reuse assembly pool objects that keep their shortest pathways of construction minimal, Fig. 2*D*. These observations suggest that MA captures more underlying structures and submolecular symmetries, while Bertz and Böttcher are sensitive to the total number of bonds. We next calculated the relative mean squared error (MSE) for simple baseline models (linear regression, logarithmic regression, power law, and polynomial regression) regressed on the weight of the molecule, the number of bonds in the molecule, the mass of the maximum peak in its spectra, and the number of peaks in its spectra (*SI Appendix*, Table S1). We note that structural elucidation is required to determine the $N_{B}$ and molecular weight (MW) of a molecule, the latter of which is not always derivable from the MS^1^ data because the parent molecule is often fragmented and a high-resolution mass spectrometer may be unavailable. For all three measures, models regressed on features that require structural elucidation (i.e., $N_{B}$ and MW) and predicted complexity better than features that do not require structural elucidation (i.e., number of peaks and maximum peak, *SI Appendix*, Table S1). This suggested that molecular complexity cannot be predicted well from simple baseline models applied directly to MS^1^ data alone, and highlighted the overall motivation for our current study (*SI Appendix*, Table S1).

It is difficult to interpret how Böttcher and Bertz score scale; for example, it is unclear what a Bertz score of 4,000.17 means relative to scores of 40.23 or 400.09. In contrast, MA has a directly interpretable scale: a molecule with an MA of 40 has a shortest pathway twice as long as a molecule with an MA of 20, see Fig. 2*A* as an example. Within the context of AT, the combinatorial spaces of physically plausible molecules in the absence of any selective bias are given by *assembly possible*, where molecules are represented by their shortest construction paths (33). With an increase in MA, the number of combinatorial construction operations leading to all physically plausible molecules increases exponentially. As an example, the molecule with an MA of 40 has a vastly larger assembly possible space compared to the molecule with an MA of 20. The exponential expansion of assembly possible also allows for interpretation of MA differences considering single construction steps a→a+1, where a quantifies an intermediate construction step, as the assembly possible expands less so with an MA from 10 to 11 as compared to an MA from 20 to 21 (Fig. 2*A*). Additionally, for an object with number of bonds $N_{b}$, the assembly index is bounded with the lower and upper bounds as ${\text{log}}_{2}(N_{b})$ and $\left(N_{b}-1\right)$ respectively. Additional constraints can be introduced, such as the number of building blocks, which can give a tighter upper bound. This suggests that for a given number of building blocks, the number of plausible molecules at any MA is finite. In contrast, for given Böttcher and Bertz scores, it is difficult to quantify the potential combinatorial spaces of molecules as they calculate complexity based on additive and entropic properties of molecular substructures instead of path-dependent features in assembly spaces. This is because the expansion of the combinatorial space of molecules cannot be interpreted intuitively with Böttcher and Bertz scores, whereas the scalable interpretability of MA has been investigated across a variety of additional contexts, Table 2.

**Table 2.** Motivation for selecting MA for life detection

Characteristic		MA	Böttcher	Bertz
a	Dependence on molecular size	Sublinear	Linear	Superlinear
b	Interpretability	High, unique construction pathway	Low, hard to interpret without context	Low, hard to interpret without context
c	Proposed for life detection	Experimental evidence of nonlife/life threshold (29)	Not currently	Not currently
d	Interpretable for individual and ensemble of molecules	Scores for both [MA (33) and JMA (34)]	Score for only individual molecules	Score for only individual molecules
e	Predictable from MS	Algorithmic, related to MS (30)	Entropic, unrelated to MS	Entropic, unrelated to MS
f	Computation time from known structure	High	Medium	Low

Comparison of three molecular complexity approaches (MA, Böttcher, and Bertz). (a–e) Five key advantages that identify MA as the leading agnostic biosignature candidate for space missions that utilize MSn. (f) Bertz scores tend to be the fastest to compute.

The same set of molecules yield substantially different ranges across the three different approaches, with maximum MA: 24, Böttcher: 911.25, and Bertz: 2,515.02, Fig. 2. Because each complexity measure captures different molecular attributes, the molecules ranked as low or high in complexity vary depending on the metric. Here, Bertz tends to classify linear hydrocarbon chains with simple or no branching structures as low in complexity, and molecules with dense, intricate bonding as high in complexity. In contrast, Böttcher evaluates the local microenvironment of atoms, ranking molecules with multiple stereocenters and conformational flexibility as more complex, while considering rigid and achiral molecules (such as symmetric cages or fullerenes) as less complex. Meanwhile, MA considers how many steps are required to form molecules, and hence it ranks heterogeneous molecules with few reusable substructures as high in complexity, and molecules that can recursively reuse substructures in their assembly pools as low in complexity.

It is noteworthy that, when normalized for size, polycyclic aromatic hydrocarbons (PAHs) are typically ranked as highly complex according to Bertz, but as having low complexity according to MA, Fig. 2. This is due to the fact that the molecular assembly in highly periodic structures scales logarithmically with the size of the molecules (or number of bonds). This is due to the presence of periodicity in the structure, and they are often observed as broad distributions, which makes them weak biosignatures. PAHs are among the most abundant types of molecules in space, believed to comprise up to 10 to 15% of the interstellar carbon (43, 44). They are also widespread (45), having been detected in interstellar dust (where stars and planets form) (46), in meteorites (47), and in the atmosphere of Titan (48). The untested “PAH world” hypothesis even proposes that PAHs facilitated the synthesis of the first RNA molecules, potentially playing a major role in the origin of life (49). Collectively, these observations underscore how different complexity scores can result in contrasting perspectives on molecules with significant astrobiological implications.

We also chose to focus on MA because it has been specifically developed for life detection methods, following experimental evidence suggesting that MA values above certain thresholds may strongly indicate the presence of life (29). If such thresholds exist for MA, they may be linked to more intuitive scalability and the statistical improbability of observing a large “copy number” of any given high MA molecule by random, undirected processes. This improbability arises not only from the large number of assembly steps required to construct the molecule, but also from the exponentially larger space of similarly complex molecules that could have been formed. We note that this copy number criterion is inherently satisfied in MS data, as modern instruments only detect molecules once a sufficiently large number of copies are present (50) (Table 2, c). Experimental work has suggested that biological samples have a wider distribution of MA values compared to abiotic samples, and that only biological samples produce MAs above a certain threshold (ca. 15) (29). We investigated how well separating the COCONUT 2.0 database (51) into two groups of molecules based on MA thresholds of between 15 and 20 could similarly separate the molecules into the same two groups based on Bertz and Böttcher scores (*SI Appendix*, Figs. S2 and S3). This analysis suggests that MA thresholds that have false positive rates (FPRs) of close to zero, based on prior experimental work, cannot be as readily translated into Bertz and Böttcher thresholds without introducing FPRs in the range of 0.17 to 0.32 (depending on the MA threshold).

**Fig. 3.**
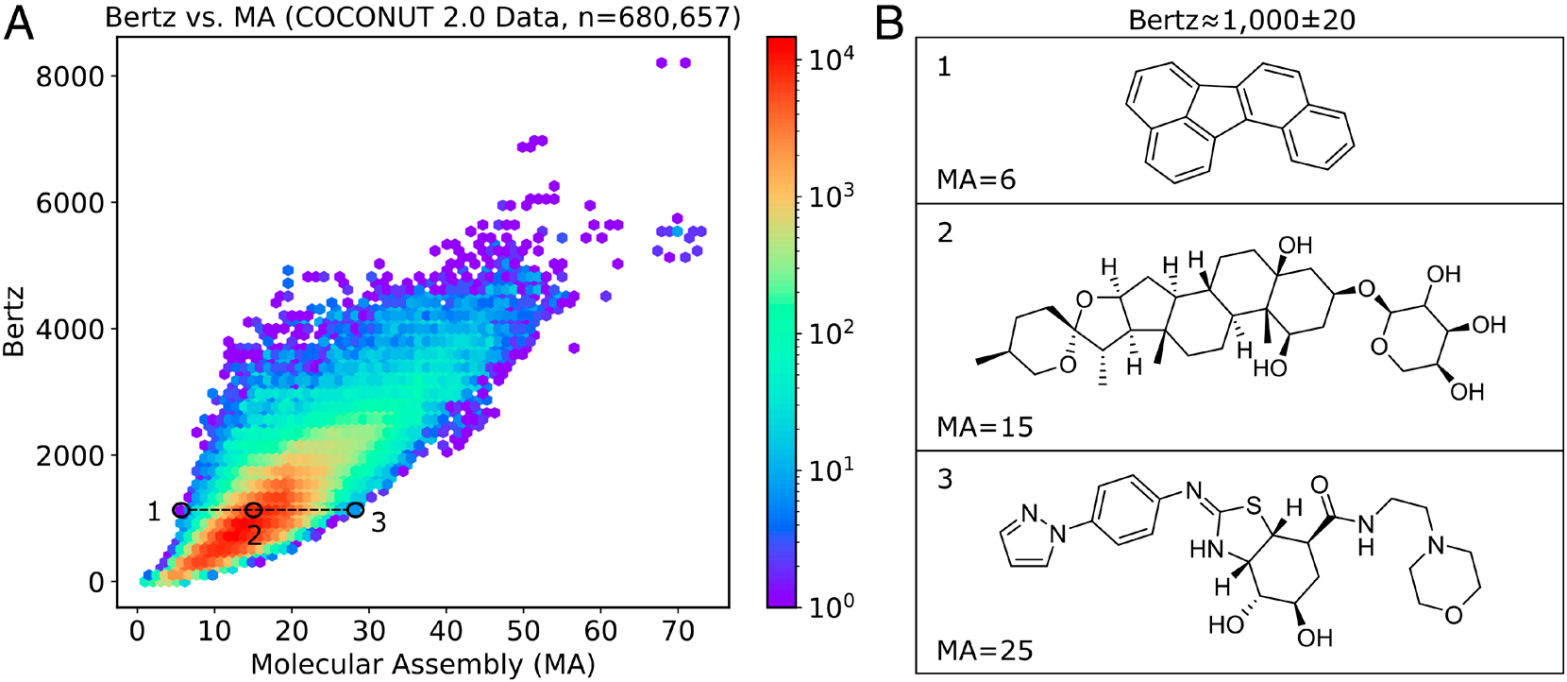
Comparing MA and Bertz scores, conditioned on constant Bertz scores. (*A*) Bertz vs. MA scores for each molecule in the COCONUT 2.0 database (51), with the color legend indicating the count of molecules on a logarithm scale. (*B*) Three example molecules with similar Bertz scores, but with either low, intermediate, or high MA scores.

The observations that reveal the relationship between Bertz and MA values for all molecules in the COCONUT 2.0 database (51) are illustrated in Fig. 3. While there is a relationship between these scores (primarily due to all complexity scores in general becoming higher with increasing molecular size), we still see significant discrepancies. For example, the three molecules shown all have the same Bertz score of around 1,000, with molecule #2 representing the average situation with an MA score of 15. However, molecule #3 has an MA of 25, which is more than four times as high than the MA score of 6 for molecule #1. This means that even though molecule #3 requires more than four times as many assembly steps to form its shortest pathway compared to molecule #1, they still obtain the same Bertz score (see the shortest pathways for these molecules drawn out in *SI Appendix*, Figs. S3 and S4).

Altogether, these example molecules in Fig. 3 illustrate that Bertz scores can be particularly high for periodic molecules with many bonds, even when their structural heterogeneity is limited. Molecule #1 [benzo(j)fluoranthene, B*j*F, which has been detected in meteorites (52)] has a Bertz score of around 1,000, which corresponds to an average MA of around 15, which would be high enough to warrant consideration as a biosignature.

However, B*j*F has a true MA of 6, which is much lower than what would constitute strong evidence of biological origins. This again illustrates that simple translation between the complexity scores is not possible. We showcase various other example molecules with strikingly different scores between complexity approaches in *SI Appendix*, Figs. S5–S9.

**Fig. 4.**
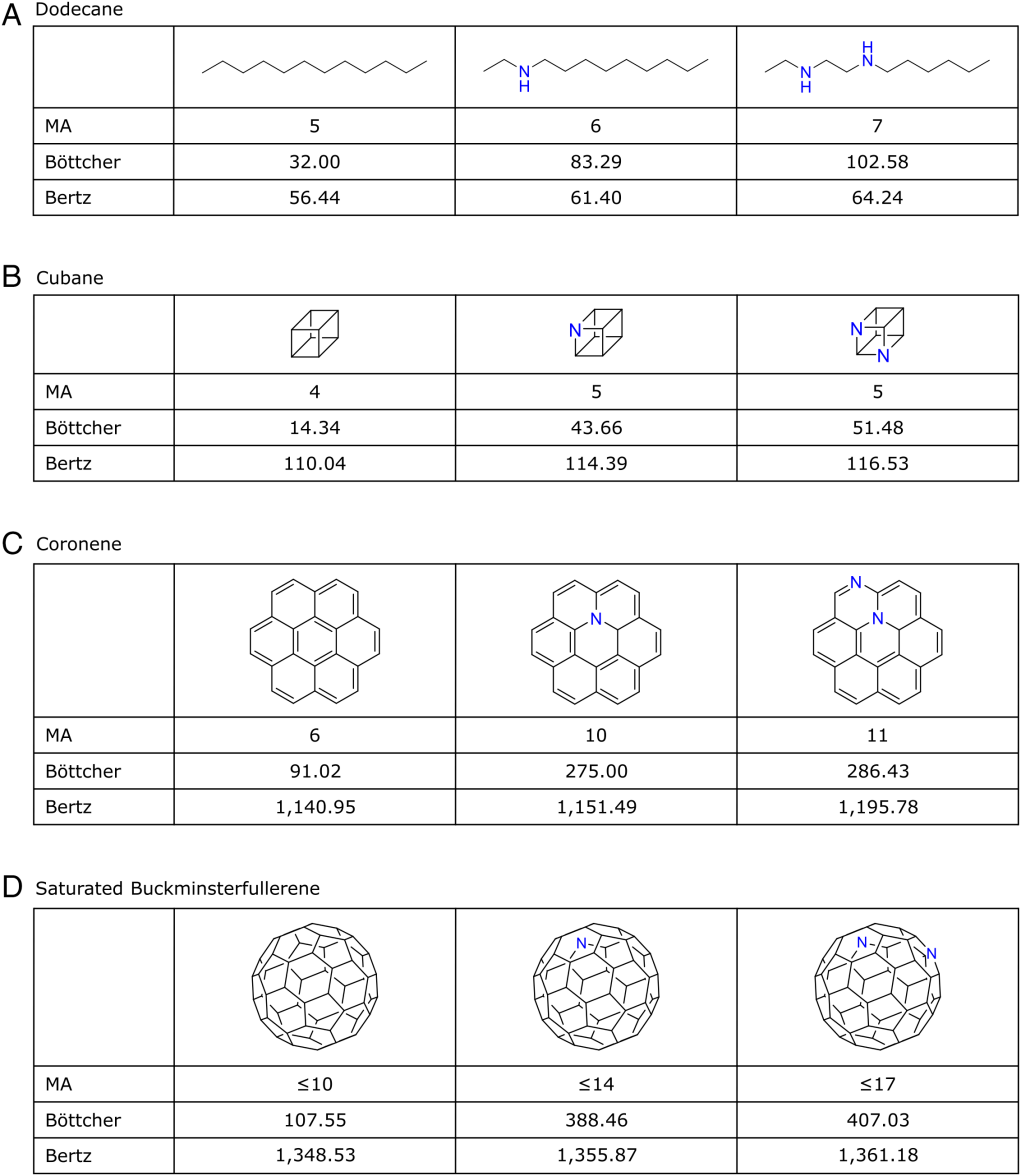
The effect of symmetry breaking on complexity values. (*A*–*D*) Highly symmetrical hydrocarbon molecules: dodecane, cubane, coronene, and saturated buckminsterfullerene. From *left* to *right*: the highly symmetrical hydrocarbon molecule, the same molecule with one nitrogen replacing one carbon, and the same molecule with a second nitrogen replacing a second carbon. We used the saturated version for buckminsterfullerene to avoid kekulization issues that would affect MA calculations (*SI Appendix*, section 1.5).

We highlight additional differences between the complexity values through a deliberate symmetry-breaking analysis, Fig. 4. For this, we start with a set of highly symmetrical hydrocarbon molecules—dodecane (the largest organic molecule discovered to date on Mars) (53), cubane, coronene [present in meteorites and the interstellar medium (ISM)] (54), and buckminsterfullerene (also present in meteorites and the ISM) (55, 56)—and then break their symmetry by adding “defect” atoms, Fig. 4. Here, Böttcher scores increase significantly, while Bertz scores and MA increase only slightly. In this demonstration, MA and Bertz are sensitive to both full and submolecular symmetries, whereas Böttcher is sensitive to the full molecular symmetry and largely unaffected by submolecular symmetries.

**Fig. 5.**
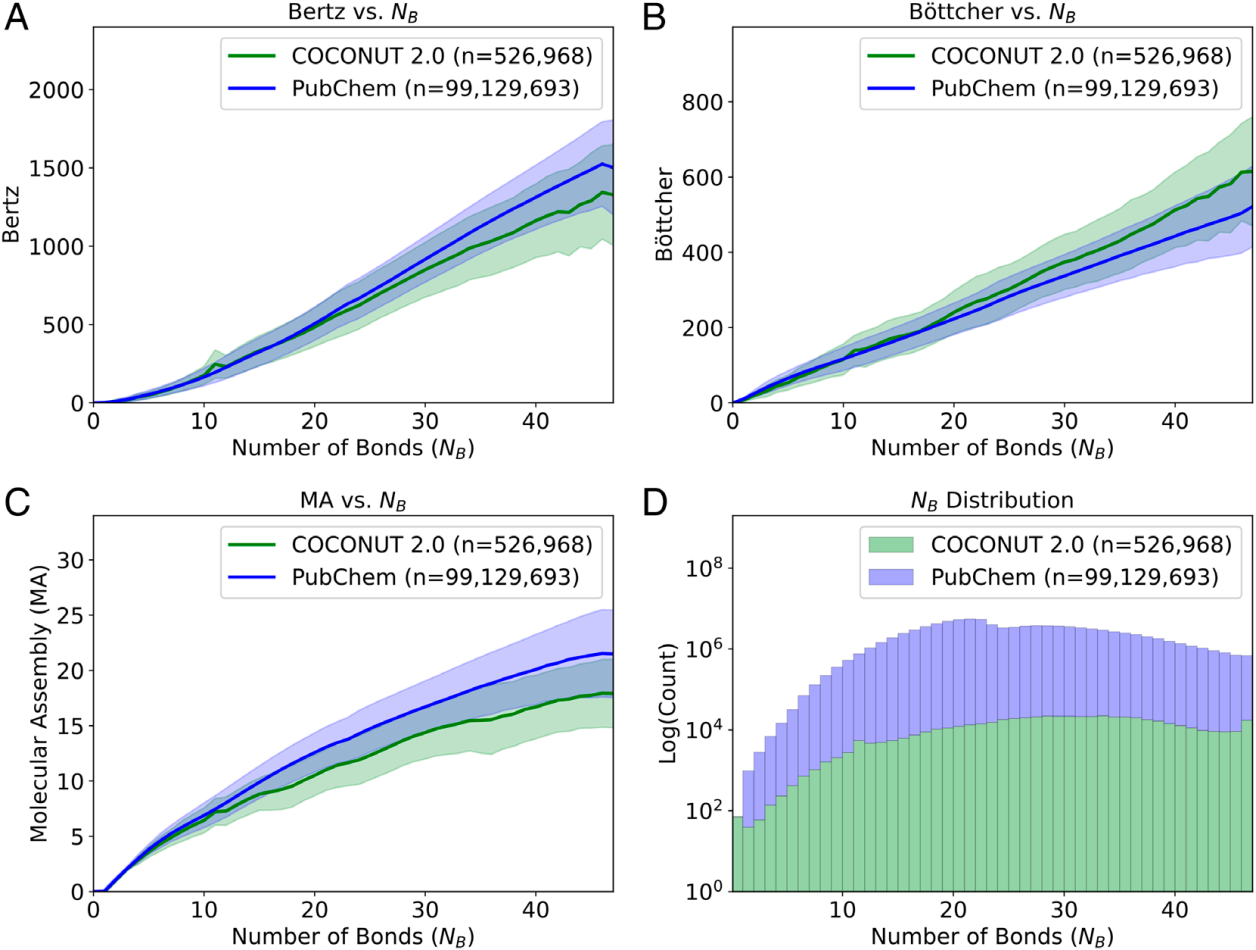
Molecular complexity growth rate per bond count between PubChem and COCONUT 2.0 molecules. Mean (solid line) and standard deviation (shaded area) of complexity scores per bond count between PubChem molecules in blue (n ≈ 100,000,000) and COCONUT 2.0 molecules in green (n ≈ 525,000) for (*A*) Bertz, (*B*) Böttcher, and (*C*) MA. (*D*) The databases had a similar distribution of bond counts in their molecules.

To further examine the role of MA in life detection, we investigated how well the three approaches distinguished between similarly sized molecules from the PubChem and COCONUT 2.0 databases (Fig. 5) (51, 57). In a broad manner, the PubChem database (57) can serve as a proxy for what molecules are possible given the environmental constraints on planet Earth, whereas the COCONUT 2.0 database (51) can serve as a proxy for what biologically derived natural product molecules are the outcome of evolution and selection given these same constraints. Fig. 5 shows that, as expected, for both databases, both the mean and variance of Bertz and Böttcher scores generally increase as *N_B_* increases. However, Bertz scores tend to be higher for PubChem molecules compared to COCONUT 2.0 molecules of the same size, whereas Böttcher scores show the opposite trend. As expected MA shows sublinear trend seen earlier in Fig. 2*D*, and higher values are seen in PubChem molecules compared to the same size COCONUT 2.0 molecules.

Intriguingly, MA appears to distinguish between the databases starting from smaller $N_{B}$ values: for example, at $N_{B}=20$, MA separates the databases such that the mean of one coincides with the standard deviation of the other (Fig. 5*C*). This is not seen in the cases of Bertz and Böttcher, where the means and standard deviations are not readily differentiable between the databases (Fig. 5 *A* and *B*). This pattern remains even when we subset the databases to only consider molecules containing carbon, hydrogen, nitrogen, and oxygen (*SI Appendix*, Fig. S11), and it remains (albeit in a reduced manner) when we examine the inverse set of molecules (i.e., those that contain at least one atom that is *not* carbon, hydrogen, nitrogen, and oxygen) (*SI Appendix*, Fig. S12). Altogether, this suggests that MA may detect differences between biological and nonbiological origins starting from smaller sized molecules. We also examined the upper-bound of MA across both databases, and found that, for each $N_{B}$ value, the largest MA from PubChem was greater than or equal to the largest MA from COCONUT 2.0 (*SI Appendix*, Fig. S13) (51, 57). The largest $N_{B}$ value that maintained the strict upper-bound of $\mathrm{MA}=N_{B}-1$ occurred for $N_{B}=14$ in the COCONUT 2.0 database, and occurred much later, at $N_{B}=24$ in the PubChem database.

Upon inspecting these unusually heterogeneous molecules, we found that PubChem contains pharmaceutical compounds that require synthetic intervention, thus allowing for complex molecules to occur, even at relatively smaller molecular sizes. These PubChem molecules that contain large MA values (especially above any $N_{B}$-matched COCONUT 2.0 molecules) can potentially be studied as “molecular technosignatures,” i.e., compounds that are unlikely to occur naturally without technological intervention (58, 59) (*SI Appendix*, Fig. S13). An additional reason we chose MA for this study was because algorithms like RecursiveMA can predict MA from MS^n^ data, given that smaller peak masses of later stages add to form larger peak masses of earlier stages in a recursive manner that parallel the joining operations of AT (30). In contrast to the more abstract joining operations of molecular graph correlates, Böttcher and Bertz depend heavily on explicitly labeled molecular properties (such as bond types, atom types, valence electrons, stereocenters, and their configurations, etc.) (31, 32), and thus require greater structural elucidation than approaches based on features more interpretable from MS data, such as the additive properties of peak masses [two m/z values of fragments can be summed together to represent a larger fragment or parent molecule (30)]. As a result, as far as we know, it has not yet been shown whether an algorithm could successfully predict Böttcher and Bertz values directly from MS data (Table 2, e).

Altogether, we developed MA for several reasons: it detects the underlying structure well, it provides interpretable scalability, it has shown experimental promise as a life detection tool, it provides measurements for both individual molecules and ensembles of molecules, and it can be measured from MS^n^ data (Table 2, a–e). For future life detection missions using MS^n^, MA scores can be calculated with the RecursiveMA algorithm. This study explores whether ML can be used to predict MA scores for missions that rely on MS^1^ data.

## Limitations of Algorithmic Prediction of MA from MS^1^ Data.

We briefly describe the RecursiveMA algorithm and demonstrate its intended uses and limitations. As mentioned earlier, RecursiveMA estimates MA from MS^n^ data (30). It accomplishes this by setting an upper limit of MA estimation based on the parent mass as a first-order approximation, and then correcting this score as overlaps in child fragments are discovered (34). In theory, complete information of all parent–child fragments in MS^n^ data with infinite resolution should allow for the near-perfect measurement of MA. However, real-world MS^n^ data provide only limited fragmentation information (due to neutral fragments, rearrangement reactions, limited recursions, loss of parent masses, and different bonds possessing different strengths). As a result, MA values are predicted well, but not perfectly, when using RecursiveMA in real-world MS^n^ data (30, 60).

While some proposed life detection missions in our Solar System will use MS^n^, many will use MS^1^ due to size and power constraints, the need for speed and efficiency, and the focus on in situ analysis. However, RecursiveMA is not designed for MS^1^ data, as such data do not include the relationship between parent and fragment ion masses across stages. In some MS^1^ data, parent ions are also lost, which further reduces the suitability of RecursiveMA (30). This is especially true in “hard” ionization techniques, such as electron ionization (EI) approaches, which will also be used in proposed life detection missions in our Solar System (*SI Appendix*, Fig. S15*A*). These limitations motivated us to create a ML model that can predict MA scores from MS^1^ data as a complementary approach to RecursiveMA that can predict MA scores from MS^n^ data.

## Evaluating ML Prediction of MA from MS^1^ Data.

**Fig. 6.**
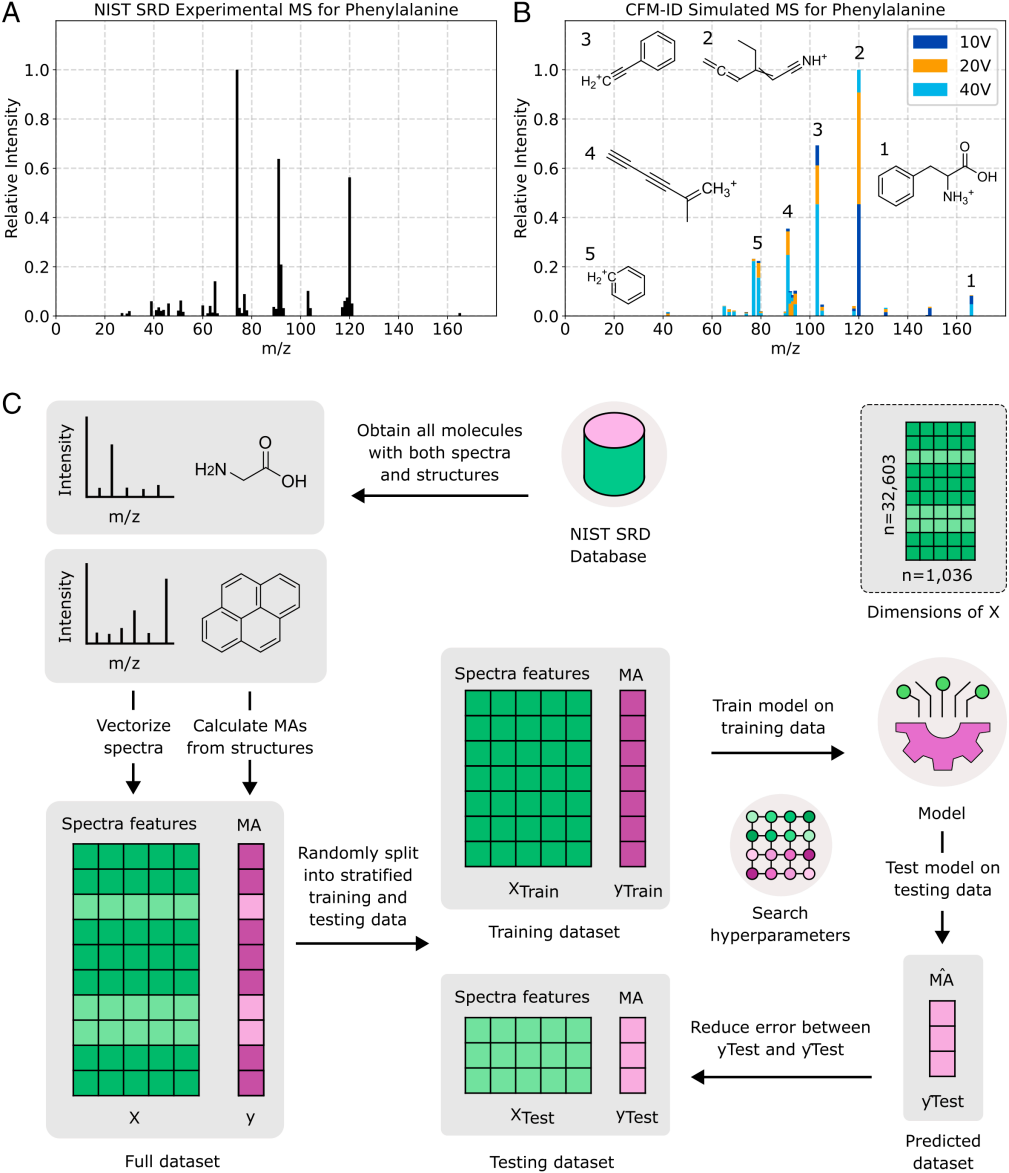
Flowchart of XGBoost model generation. (*A*) Experimental MS of phenylalanine from the NIST SRD database (usually MS^1^ EI-GCMS 70 eV). (*B*) Simulated MS of phenylalanine from the CFM-ID database [MS/MS (MS^2^) ESI] at 10 eV, 20 eV, and 40 eV. Simulated data provided fragment identification and allowed us to determine differences in m/z and intensity of peaks based on collision energy. (*C*) We obtained all molecules in the NIST SRD data that contained both MS^1^ and an elucidated structure. The explanatory variable was the vectorized MS^1^ data, where each vector index contained the peak intensity at the corresponding m/z value, with intensities normalized between zero and unity. The response variable was the true MA scores. We randomly split our full data into training (70%) and testing (30%) data using a stratified option that ensured equal distributions of true MA scores. We trained our XGBoost model on the training data with an extensive hyperparameter grid search and selected the model that minimized the relative MSE between the true and predicted MA scores of the testing data.

We assessed whether ML could allow for MA prediction from MS^1^ data (Fig. 6 *A* and *C*). For this, we trained an XGBoost (42) model on all molecules with MS^1^ data in the NIST SRD database (35), after removing a small subset of molecules that were unsuitable for analysis (*SI Appendix*, Fig. S1). We found that our model reduced the relative MSE by three-fold (from 0.12 to 0.04) as compared to the best-performing baseline model (*SI Appendix*, Table S2).

**Fig. 7.**
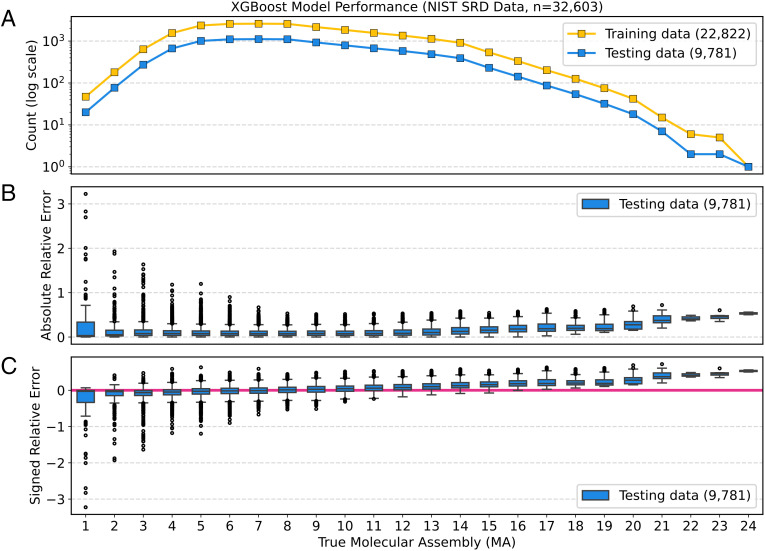
XGBoost model performance on the NIST SRD data. (*A*) Count of molecules per MA bin for the training and testing data, (*B*) absolute relative error (calculated as $\left|y-\hat{y}\right|/y$), and (*C*) signed relative error (calculated as $\left(y-\hat{y}\right)/y$), where y equals the true MA score and $\hat{y}$ equals the predicted MA score. Comparing *A* and *B*, we see an anticorrelation between the absolute relative errors and the count of molecules for each MA score in the data. As *C* shows, low MA molecules tended to be overpredicted (leading to a negative signed prediction error) and high MA molecules tended to be underpredicted (leading to a positive signed prediction error). This trend revealed that our model was conservative in mitigating false positive assignment of life detection (i.e., was unlikely to overestimate the MA scores of high MA molecules).

We observed that molecules with low MAs tended to be overpredicted and molecules with high MAs tended to be underpredicted (Fig. 7). The molecules with low MAs were likely overpredicted due to the strict lower bound of zero for possible MA. Importantly, the underprediction of high MA molecules ensured that our model remained conservative, mitigating the risk of falsely categorizing molecules as stronger life indicators than they might truly represent. We also performed various quality checks and confirmed that our XGBoost model demonstrated the ideal learning curve (*SI Appendix*, Fig. S14), implying that our model was neither under- nor overfitting to the training data. Next, we evaluated whether our well-performing XGBoost model, trained on the NIST SRD database (35), could generalize to other datasets. For this, we obtained all EI-B MS^1^ data (electron ionization on a magnetic sector instrument) from the MassBank repository (61) and removed any molecules that had already been included in the NIST SRD database. Then, we applied our XGBoost model from the NIST SRD data on the MassBank EI-B MS^1^ molecules, resulting in a relative MSE of 0.07. This still demonstrated an improvement over the best power law model fitted to molecular weight (0.12) (*SI Appendix*, Table S2). We noticed that MassBank allows users to fill out more than 100 metadata fields for their uploaded samples, with many fields encompassing spectral information, such as instrument type, run time, and collision energy. While our XGBoost model confirmed that ML can predict MA reasonably well from MS, we wished to investigate how these MS instrumental parameters affect the accuracy of this approach. In the next section, we investigate this question in detail by using simulated MS data.

## Determining Instrument Effects on ML Error Using Simulated MS.

We generated theoretical MS data for the same molecules from the NIST SRD database using the Competitive Fragment Modeling IDentification (CFM–ID) version 4 software (62). For each molecule, we generated three Electrospray Ionization (ESI) Quadrupole Time of Flight (QToF) [M + H]+ + MS^2^ (MS/MS) data, with low (10 eV), medium (20 eV), and high (40 eV) collision energy levels. The CFM–ID software was able to produce spectra for all the NIST SRD molecules used in our main study, except for 14 molecules, which we therefore excluded.

We trained three XGBoost models (one for each energy level) with the same approach we used for the experimental NIST SRD dataset (Fig. 6 and *SI Appendix*, Table S4). Compared to the experimental data, the simulated data further reduced the relative MSE to values of 0.033 (for the 10 eV-only data), 0.031 (for the 20 eV-only data), and 0.031 (for the 40 eV-only data), yielding a fourfold (0.12 to 0.03) reduction in our error metric compared to the best-performing baseline model (diagonal cells in *SI Appendix*, Tables S2 and S4). It is likely that simulated MS data mitigate the inherent noise in experimental data, enhancing the predictability of MA scores, although it is not entirely a fair comparison, as these two datasets had other differences (the NIST SRD data were MS^1^ that used EI, whereas the CFM–ID data were MS^n^ that used ESI).

We also trained an XGBoost model on split-energy data, where one-third of the MS data was from each of the three energy levels respectively. Our split-energy model resulted in a relative MSE of 0.035, which was larger than any of the single-energy models (diagonal cells in *SI Appendix*, Table S4). These results confirmed reduced MA prediction accuracy when the data were acquired using inconsistent instrument parameters. These observations were expected as we had already confirmed that different energy levels capture different information about the MS fragments (Fig. 6*C* and *SI Appendix*, Figs. S15–S17).

If it is straightforward to measure MA with MS, then it would hypothetically be more powerful to combine information across various energy levels to simultaneously capture as many different subsets of MS fragments as possible. To investigate this, we trained an XGBoost model on integrated-energy data, where each sample contained concatenated spectral data from all three energy levels (10 eV, 20 eV, and 40 eV), providing a combined representation. As expected, our integrated-energy model performed the best of the five models, achieving the lowest relative MSE of 0.029 (diagonal cells in *SI Appendix*, Fig. S18 and Table S4). The XGBoost model trained on each of our five models (diagonal cells in *SI Appendix*, Table S4) was then evaluated with the test sets from the other four models (nondiagonal cells in *SI Appendix*, Table S4). For each XGBoost model trained on a single-energy level, the relative MSE increased when the testing set came from one of the other two energy levels or the mixed-energy level, with an almost doubling of error when the model trained on the 40 eV data was tested on the 10 eV data (0.031 to 0.060) (*SI Appendix*, Table S4). For our XGBoost model trained on the integrated-energy level, we observed a more than 2.3-fold increase in error when tested on the 10 eV data (0.029 to 0.067) (*SI Appendix*, Table S4). These findings highlight the importance of consistent instrumental parameters between the training and testing data for optimal prediction of MA from MS datasets.

Even though our model performs much better than baseline models, we conducted several post hoc analyses to determine the underlying causes of over- and underprediction of MA in our XGBoost models (*SI Appendix*, section 4). We found that heavier molecules that break into an unusually large number of fragments (such as molecules with long hydrocarbon chains) tend to increase the chance of an overprediction, while heavier molecules that do not break at all or only break into an unusually small number of fragments (such as molecules with fused benzene rings and steric hindrance) tend to increase the chance of an underprediction (*SI Appendix*, Figs. S19–S21). In some cases, molecules that were substantially underpredicted by the model passed through the mass spectrometer without undergoing detectable fragmentation, yielding spectra with only a single peak (*SI Appendix*, Fig. S21).

Hence, it seems that our XGBoost model itself does a suitable job of learning how to predict MA scores from MS data, as corroborated by our smooth and convergent learning curves (*SI Appendix*, Fig. S14). Instead, the current limitations are not so much due to the model itself, but due to MS instrumentation constraints. Given that our model performed best with the integrated-energy data in *SI Appendix*, Table S4, we believe it is likely that multimodal approaches could improve our model even further. For example, a multimodal model that incorporated MS data from energy levels even harsher than 40 eV could potentially allow for the fragmentation of molecules that are otherwise too stubborn to break at all in lower energy levels, decreasing their tendency for underprediction (*SI Appendix*, Fig. S21). Altogether, it is likely our XGBoost model would effectively learn and benefit from such multimodal MS information.

## Conclusions

In this work, we presented a proof-of-concept demonstrating the potential use of ML to predict the MA molecular complexity as a biosignature from in situ mass spectrometers during future space missions. Although the application of ML in life detection remains a nascent endeavor, it holds significant promise (63). Our work builds upon recent advancements in the field, complementing ongoing efforts that underscore the growing utility of ML in the search for alien life (64, 65). Additionally, we used simulated data to highlight how instrumental parameters in MS can influence fingerprint reproducibility, potentially diminishing the effectiveness of training sets. Our study emphasizes the need for well-standardized, well-documented MS databases to optimize their use in ML applications.

Our proof-of-concept considered the MA values of individual molecules. Implementation of this metrology on other planets would require chromatographic separation of individual molecules before fragmentation in the mass spectrometer. Although XGBoost substantially improves the prediction of MA from standardized MS^1^ data, robust application of the approach will require standardized and calibrated datasets, careful control of instrumental parameters, and consideration of the informativeness of the acquired spectra. Nonetheless, it could be of interest to conduct a similar proof-of-concept that considers the JMA indexes of ensembles of molecules. In future studies, it may also be valuable to infer MA from NMR data using ML (30). If it is true that NMR approaches involve less variability in instrumental parameters compared to MS (66), then this could lead to better standardized data and, consequently, improved model performance. Although NMR is less likely to be deployed in proposed space missions due to its size, weight, and power consumption, such a project could still provide meaningful insights. For example, it could be useful for predicting MA as a biosignature in extraterrestrial samples deliberately returned to Earth (67), or in extraterrestrial samples that arrive naturally on Earth, such as the ALH84001 meteorite (68). Inferring MA from infrared (IR) data using ML is another area of interest, especially in the case of investigating biosignatures in exoplanetary atmospheres. In some scenarios, it could be worthwhile to employ multimodal approaches that combine information from MS, NMR, and/or IR data to further improve the predictive power of molecular complexity when structural elucidation is difficult or impossible (30, 69).

## Methods

For the experimental data, we used all molecules with MS^1^ data on the NIST Chemistry WebBook (SRD 69) (35). We generated their corresponding theoretical MS^n^ data using the CFM-ID version 4 software (62), with low (10 eV), medium (20 eV), and high (40 eV) energy levels. Our XGBoost model (42) was developed with the Python package xgboost using a loss function of relative MSE, calculated as 1n∑i=1ny^i-yiyi2. We calculated MA scores using the assemblyCPP version 5 software (70), Böttcher scores using code from the Forli Lab at Scripps Research (https://forlilab.org/code/bottcher/) as $\sum_{i}d_{i}e_{i}s_{i}{\text{log}}_{2}\left(V_{i}b_{i}\right)-\frac{1}{2}\sum_{j}d_{j}e_{j}s_{j}{\text{log}}_{2}\left(V_{j}b_{j}\right)$, and Bertz scores using the BertzCT function in the RDKit package (71). Complete details of all methods are described in *SI Appendix*.

## Supplementary Material

Appendix 01 (PDF)

## Data Availability

All relevant data including the molecular data have been deposited in Zenodo (10.5281/zenodo.20586263) (72). The calculator (70) used for the assembly index is available at https://github.com/croningp/assemblycpp-v5. All other data are included in the manuscript and/or *SI Appendix*.
