# Supplementary material for: Molecular assembly as a universal biosignature measurable by mass spectrometry
Source: Proc Natl Acad Sci U S A. 2026 Aug 10;123(33):e2619198123. doi: 10.1073/pnas.2619198123 (PMC13486590; doi:10.1073/pnas.2619198123)
Supplement: Supplementary file 1 [file pnas.2619198123.sapp.pdf]

Supplementary Material

Molecular assembly as a universal biosignature measurable by mass spectrometry

Lindsay A. Rutter†, Abhishek Sharma†, Ian Seet, David Obeh Alobo, An Goto, and Leroy Cronin*

[1]School of Chemistry, University of Glasgow, University Avenue, Glasgow G12 8QQ, UK.

*Corresponding author Email: Lee.Cronin@glasgow.ac.uk †Equal contribution

Table of Contents

1. Methods....2
   1.1 Experimental mass spectrometry data acquisition....2
   1.2 Simulated mass spectrometry data acquisition....2
   1.3 Removal of invalid molecules....2
   1.4 Molecular complexity calculations....3
   1.5 Molecular symmetry breakage analysis....6
   1.6 ML model development....7
   1.7 Figure generation....9
2. Rationale for selecting MA as molecular complexity score....9
3. Evaluating ML prediction of MA from MS^1 data....25
4. Determining instrument effects on ML error using simulated MS....28
5. Identifiers of example molecules....39
6. Supplementary file references....44

1. Methods

1.1 Experimental mass spectrometry data acquisition

To train and test our model, we used NistChemPy, a Python Application Programming Interface (API), to obtain all molecules with spectrometry data on the NIST Chemistry WebBook (NIST Standard Reference Database (SRD) 69)[1]. We used the MassBank dataset (2024.06 release)[2,3].

1.2 Simulated mass spectrometry data acquisition

We generated theoretical mass spectrometry data for the same molecules from the original NIST SRD database[1] using the Competitive Fragment Modeling IDentification (CFM-ID) version 4 software[4]. The spectra are Electrospray Ionization (ESI) Quadrupole Time of Flight (QToF) [M+H]+ tandem mass MS/MS (MS^2) spectrometry, and we generated three spectra for each molecule, with low (10eV), medium (20eV), and high (40eV) energy levels. The CFM-ID software was able to produce all three spectra for all the NIST SRD molecules that we used in our main study, except for fourteen molecules that did not produce any simulated spectra. We therefore excluded these fourteen molecules.

1.3 Removal of invalid molecules

We removed several molecules in the datasets that were unsuitable for analysis. First, we removed stereoisomers from the data to eradicate redundancies. We then removed molecules inappropriate for MA calculations: we removed disjointed molecules because their MA scores cannot be calculated since they represent disconnected graphs and MA calculation requires connected graph representations of the molecules. We also removed molecules with transition metals and post-transition metals because they can form compounds with multi-center bonds such as ferrocene; these molecules may have a poorly defined bonding scheme. We also

removed deuterated and tritiated molecules as MA calculations do not consider isotope differences. We removed molecules with an MA score of zero as these molecules have only one bond and are trivial to identify (Supplementary Fig. 1). Lastly, we removed molecules that were stereoisomers of each other to avoid redundancies.

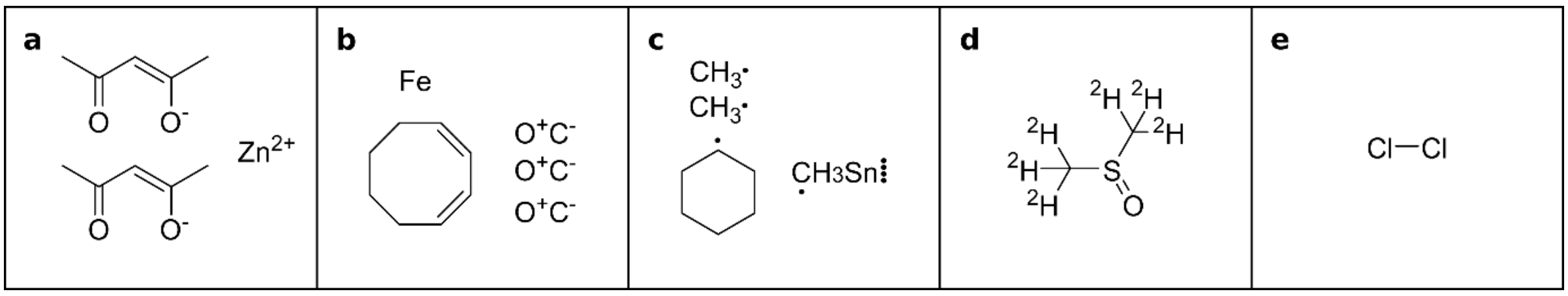

Supplementary Fig. 1: Examples of molecules removed as unsuitable for the analysis. We removed **a,** disjointed molecules, **b,** molecules with transition metals, **c,** molecules with post-transition metals, **d,** deuterated molecules (and tritiated molecules), and **e,** and molecules with an MA score of zero.

1.4 Molecular complexity calculations

Molecular Assembly (Assembly Index)

Molecular Assembly is defined by the minimal number of steps required to construct a molecule using molecular bonds as building blocks. We calculated MA scores using the assemblyCPP version 5 software.[5] We ensured molecules had hydrogen atoms removed before calculating the number of bonds and the molecular complexity scores. For most molecules, we used the "exact" method, which guarantees correct MA calculation if the process completes before the specified timeout. For the small fraction of molecules (e.g. 0.009% and 1.5% of the molecules for PubChem and COCONUT 2.0 databases, respectively) that timed out with the "exact method", we used the "breadth-first" approximation (BFA) method, which estimates MA calculation. The BFA method returns a reasonable estimation of MA scores (i.e. either the exact/true MA score or a slight overestimation). This process ensured that we obtained the

exact MA score for almost all our molecules, and a reasonable estimation of MA scores for only a small fraction of molecules.

Computations were performed on a Linux server at the University of Glasgow with dual AMD EPYC^{TM} 7763 64-core processors (128 physical cores, 256 threads total, max clock speed 3.53 GHz), 503 GB of RAM, and over 10 TB of storage. To calculate molecular complexity scores for millions of molecules, we used joblib.Parallel to process batches of molecules in parallel. For most molecules, we used the "exact" algorithm (algoType=0) with a maximum run time of 1,200,000,000 microseconds (20 minutes). For this, we issued the command:

```
assemblyCpp -algoType=0 -runTime=1200000000
```

For the small subset of molecules that exceeded the allocated maximum run time of 20 minutes, we used the "breadth-first" approximation algorithm (algoType=1) with the same input parameters (i.e. the same run time of 20 minutes and the same default parameters). For this, we issued the command:

```
assemblyCpp -algoType=1 -runTime=1200000000
```

With the assemblyCPP software, two files are outputted: an "Out" file and a "Pathway" file. The "Out" file contains the MA score and the time to completion (in microseconds). The "Pathway" file contains the resulting shortest pathway of construction found to derive the outputted MA score.

Böttcher Index

The Böttcher complexity index is a molecular complexity index that assigns a single numerical value to a molecular structure by summing the complexity contribution for each atom's localised chemical environment. We calculated Böttcher scores using code from the Forli Lab at Scripps Research (https://forlilab.org/code/bottcher/). As some of our molecules were quite large and highly symmetrical, we increased several input parameter values (such as "automorp_memory_maxsize") to avoid memory limit issues for highly symmetrical molecules. In this case, we were able to obtain Böttcher scores for nearly all our molecules, with only a small fraction timing out. While the original paper did not provide code for Böttcher score calculations, the calculator from the Forli Lab attempts to translate into code the main equation proposed in the original paper[6]:

$$C_m = \sum_i d_i e_i s_i log_2(V_i b_i) - \frac{1}{2}\sum_j d_j e_j s_j log_2(V_j b_j) \quad (1)$$

where C_m defines the complexity of the molecule. The term $log_2(V_i b_i)$ is the main descriptor of each atom i, and the additional parameters d_i, e_i, and s_i. In general, the variable V_i provides the number of valence electrons and b_i describes the total number of bond orders of all bonds to non-hydrogen atoms. The term d_i describes the number of chemically non-equivalent bonds to non-hydrogen atoms, e_i quantifies the diversity of non-hydrogen elements or isotopes in the local environment of atom i, and s_i gives the number of isomeric possibilities. The complexity factor of each atom i is quantified as the product $d_i e_i s_i log_2(V_i b_i)$. To account for molecular symmetry, half of the contribution from chemically equivalent positions j is subtracted. The overall calculation of C_m then sums up the information content in the chemical microenvironment of each atom in the molecule.

Bertz Index

The Bertz complexity index is a topological descriptor of the complexity of a molecule that uses a combination of graph theory and information theory. The total complexity is defined as a sum of two different contributions: Skeletal Complexity $C(\eta)$ and Elemental Diversity $C(E)$. Skeletal Complexity measures the complexity of the molecular skeleton based on the connectivity between the atoms. It is calculated using pairs of adjacent bonds, also referred to as connections (η). Elemental Diversity measures the complexity arising from how various heteroatoms are distributed in the molecular structure. The skeletal complexity is defined as $C(\eta) = 2\eta \log_2(\eta) - \sum_i \eta_i \log_2(\eta_i)$, and hence the Bertz index becomes $C_T = C(\eta) + C(E)$. We calculated Bertz scores using the BertzCT function in the RDKit package[7]. The function attempts to quantify the complexity of molecules with the Bertz metric introduced in the original paper[8].

1.5 Molecular symmetry breakage analysis

In the comparative analysis of molecular symmetry breakage, we used saturated versions for buckminsterfullerene to avoid kekulization issues that would affect MA calculations (Fig. 4c-d). This is because kekulization is arbitrary and the seeded reference will ambiguously change when nitrogens are added. We calculated MA scores for the first three molecules using the "exact" algorithm of assemblyCPP with the same code shown earlier in Section 1.4. For the fourth molecule (buckminsterfullerene), we calculated its MA score using the "breadth first" approximation algorithm of assemblyCPP. Although the "breadth first" approximation algorithm of assemblyCPP provides near-exact MA score estimations, as we were only focusing on one molecule, we used a slightly more computationally expensive variation of the code shown earlier in Section 1.4; namely, we increased the 'searchDepth' parameter to a value

of 4 and we increased the maximum run time to a value of 12,000,000,000 miroseconds (200 minutes).

```
assemblyCpp -algoType=1 -searchDepth=4 -runTime=1200000000
```

The SMILES identification of all 12 molecules used for the symmetry breakage analysis are provided in Supplementary Section 5.

1.6 ML model development

For our machine learning models, we used a random seed to divide 2/3 of the data for the training set and 1/3 of the data for the testing set. We stratified the split to ensure that the distribution of true MA scores was comparable between the training and testing datasets. The MS^1 data contained integer resolution of one (i.e. contained intensity values for integer values of mass to charge (m/z) ratios), and we normalized the raw intensity values to a range between zero and one. We converted each spectrum into a vector where each index represented the intensity value for m/z values between one and 1,036 (the largest m/z value throughout the entire database). Our input into the model then contained a vectorized form of each MS^1 data, where the vector was of length 1,036 and contained values between zero and one (see Fig.6 from the main document).

We initially evaluated three ML algorithms: multi-layer perceptron (MLP), random forest (RF), and XGBoost. We used the Python library scikit-learn (version 1.6.1) to implement models for MLP and RF, evaluating both classification and regression approaches (MLPClassifier, MLPRegressor, RandomForestClassifier, and RandomForestRegressor)[9]. We used the xgboost python package (version 2.1.4) to implement models for both classification and regression

approaches. We investigated these models with their raw peak intensities (normalized between zero and unity), with logarithm of the raw peak intensities (normalized between zero and unity), and with binary peak intensities (either unity for peak present, or zero for peak not present). We found that XGBoost consistently performed better than MLP and RF. We chose to use regression because even though MA scores can only be integer values, they represent ordered quantities, not distinct categories without order. In general, we did not find that peak intensity preparation made a consistently clear difference in model performance. As previously mentioned, we decided to keep raw peak intensities (normalized between zero and unity), and we had two main reasonings for this decision. First, raw peak intensities allow for better discriminatory power, as some structurally related compounds (like isomers) may have the same fragment m/z values but different peak intensities. Hence, binarizing these peak intensities causes structurally related compounds to possess the exact same MS. Second, isotopic ratios are revealed in peak intensities: for instance, chlorine exists with natural abundances of ~75.8% ^{35}Cl and ~24.2% ^{37}Cl, bromine exists with natural abundances of ~50.7% ^{79}Br and ~49.3% ^{81}Br, and carbon exists with natural abundances of ~98.9% ^{12}C and ~1.1% ^{13}C.

We performed an extensive grid search to optimize model hyperparameters. Our XGBoost model[10] on the NIST SRD data was developed using the following hyperparameter values: max_depth (maximum depth of a tree) = 10, eta (learning rate) = 0.007, lambda (L2 regularization) = 75, subsample (subsample ratio of the training instances when constructing each tree) = 1, colsample_bytree (subsample ratio of columns when constructing each tree) = 0.3, gamma = 0, and min_child_weight (minimum sum of instance weight needed in a child) = 2. Our loss function was relative mean squared error (MSE), which was calculated as $\frac{1}{n}\sum_{i=1}^{n}\left(\frac{\hat{y}_i - y_i}{y_i}\right)^2$, where y_i is the true MA score and $\hat{y}_i$ is the predicted MA score from the model.

We chose relative MSE because it provides an intuitive measure of how large the error of the model is relative to the actual values of the data, rendering it easier to interpret in practical terms and allowing for better comparison between datasets.

1.7 Figure generation

High-quality drawings of molecules were generated with ChemDraw[11] Suite 23.1.2, and most figure components were generated with the Python packages Matplotlib[12] and seaborn[13]. Final figure panels were assembled and arranged using Inkscape 1.1[14].

2. Rationale for selecting MA as molecular complexity score

In this section, we use various methods to identify MA as the most suitable candidate for agnostic biosignature detection, based on its interpretability. We calculated the relative mean squared error (MSE) for simple baseline models (linear regression, logarithmic regression, power law, and polynomial regression) regressed on the weight of the molecule, the number of bonds in the molecule (N_B), the mass of the maximum peak in its MS^1 data, and the number of peaks in its MS^1 data (Supplementary Table 1). We used a similar pipeline to what we used in the end for our XGBoost machine leaning model: for example, we split the data into 2/3 training and 1/3 testing subsets and stratified the response variable to ensure equal representation between the subsets (see Fig. 6 in the main paper). For all complexity metrics, N_B provides the best predictive power: given that quantification of N_B requires structural elucidation of the molecule, this finding suggests that molecular complexity is much better predicted with more information (i.e. structural elucidation) than simply with applying baseline models to the MS^1 data. This again motivates our project to use ML to determine how well we can infer the

molecular complexity of alien molecules (i.e. unknown molecules for which we cannot determine their structures) directly from MS[1] data.

In the case of MA, N_B fits best with a logarithmic fit, where Böttcher and Bertz fits best with a power law (Supplementary Table 1). This suggests that Böttcher and Bertz scores have greater sensitivity for N_B and produce exponentially larger values as N_B increases, whereas MA scores are less sensitive to N_B and demonstrate a diminishing return with each additional bond producing less of an impact on the score. This finding corroborates what we saw in Table 1, the mathematical approach that determined that MA has sublinear dependence with N_B, Böttcher has an approximately linear dependence with N_B, and Bertz has a superlinear dependence with N_B.

Molecular Assembly (MA)	Linear	Logarithm	Power Law	Polynomial
Molecular Weight	0.15	0.13	**0.12**	0.12
Number of Peaks	0.36	0.33	**0.25**	0.35
Maximum Peak	0.15	0.13	**0.12**	0.12
Number of Bonds	0.09	0.10	**0.06**	0.06

Böttcher	Linear	Logarithm	Power Law	Polynomial
Molecular Weight	0.55	0.57	**0.48**	0.53
Number of Peaks	1.05	1.09	**0.72**	1.08
Maximum Peak	0.60	0.60	**0.53**	0.57
Number of Bonds	0.24	0.43	**0.20**	0.24

Bertz	Linear	Logarithm	Power Law	Polynomial
Molecular Weight	5.82	10.33	**4.00**	5.15
Number of Peaks	14.36	10.71	**5.45**	15.81
Maximum Peak	5.89	10.91	**4.42**	5.48
Number of Bonds	0.41	24.93	**0.29**	0.63

Supplementary Table 1: Predicting molecular complexity metrics with simple models. Relative mean squared error of simple models (linear, logarithmic, power law, and second-degree polynomial) for three molecular complexity metrics (MA, Böttcher, and Bertz) regressed on number of bonds (N_B), molecular weight, number of peaks, and maximum peak. The best-fitting model for each row is shown in bold and the best-fitting model overall is highlighted in green.

Of the three complexity metrics, we believe MA is the only one to have been proposed as a life detection tool, with experimental evidence suggesting that it is unlikely for a molecule with an MA score of approximately ≥15 to come from non-biological sources[15]. In other words, based on this experimental evidence, categorizing a molecule with MA≥15 as coming from biology correlates to a false positive rate (FPR) of near-zero. As a result, we wished to quantify any changes in the FPR if one were to simply translate MA scores to their corresponding Bertz and Böttcher scores and attempt to apply the corresponding non-life/life threshold. To achieve this, we used all molecules in the COCONUT 2.0 database[16], and applied a supervised binary classification via logistic regression, coupled with a Receiver Operating Characteristic (ROC) analysis, to find the corresponding thresholds of Bertz and Böttcher scores that best separates the molecules above and below a certain MA score threshold (Supplementary Figs. 2 and 3).

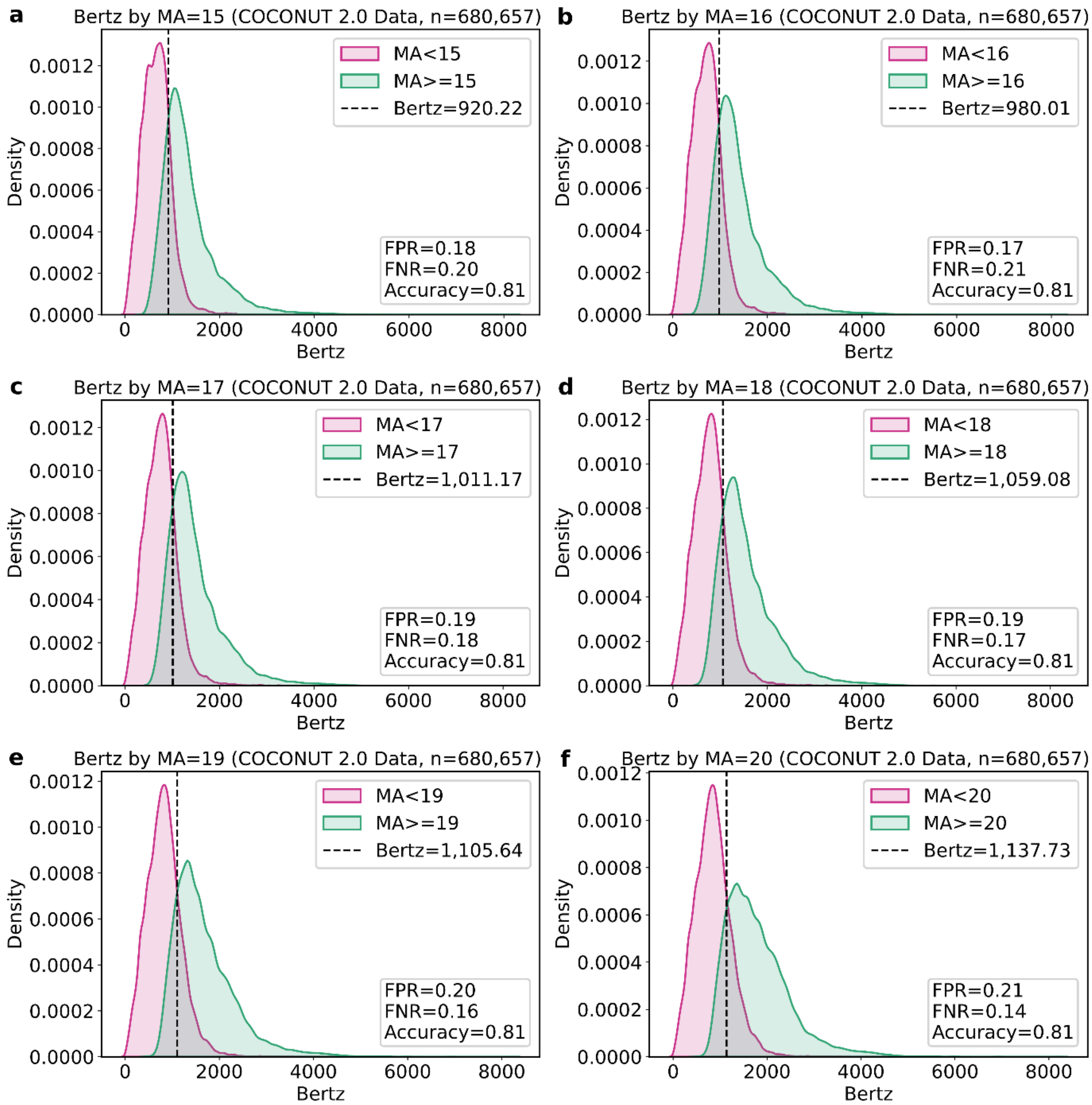

Supplementary Fig. 2: False-positive and false-negative rate evaluation of Bertz scores split by MA thresholds. Molecules from the COCONUT 2.0 database split into two groups based on a given MA threshold: **a,** MA=15, **b,** MA=16, **c,** MA=17, **d,** MA=18, **e,** MA=19, and **f,** MA=20. Then, the false-positive rate (FPR), false-negative rate (FNR), and overall accuracy were calculated regarding how well the Bertz scores could split the data into the same two groups based on the corresponding Bertz threshold for that given MA score (indicated by the vertical dotted black line). FPR values ranged from 0.17 to 0.21, FNR values ranged from 0.14 to 0.21, and accuracy values hovered at 0.81.

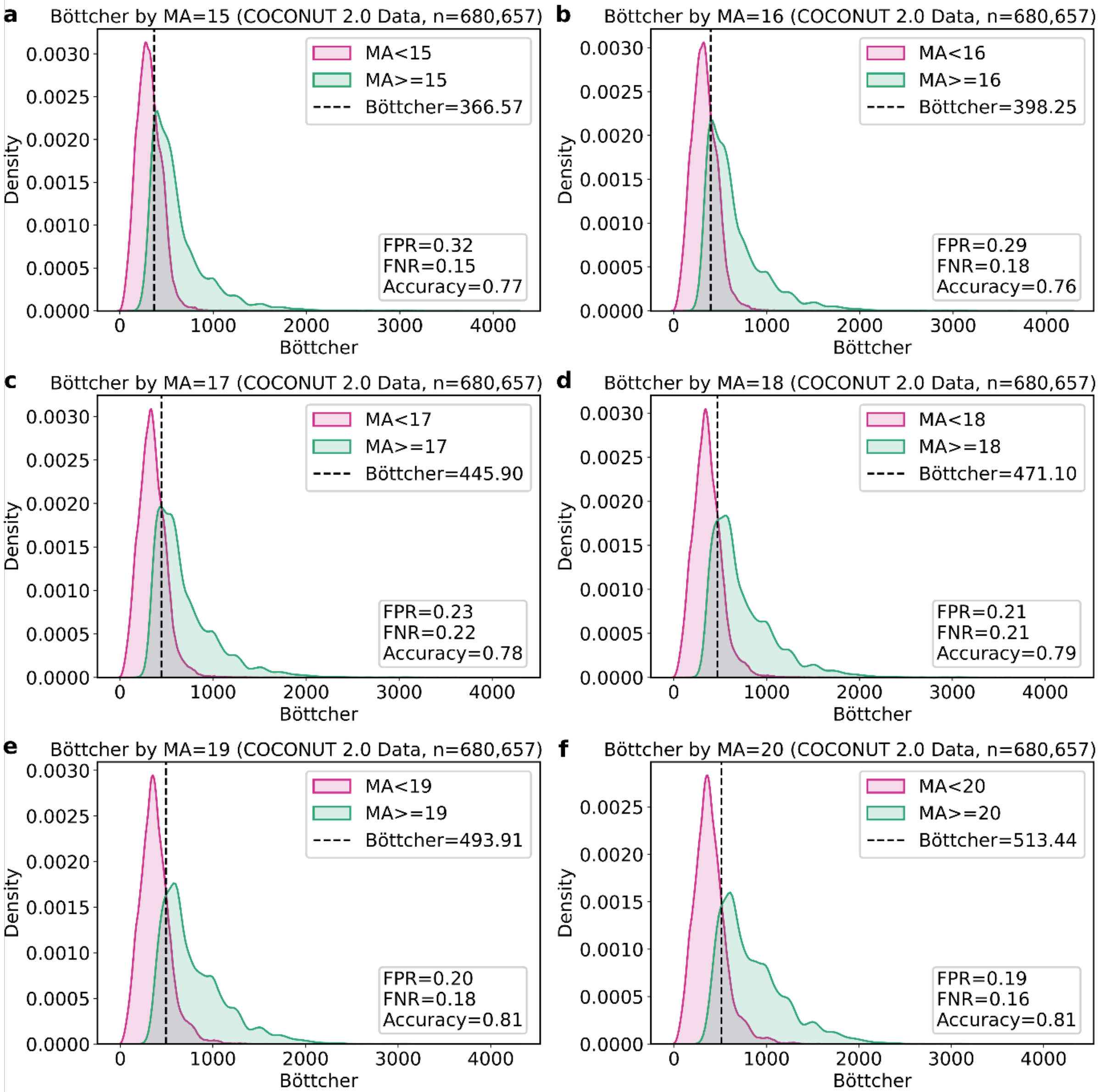

Supplementary Fig. 3: False-positive and false-negative rate evaluation of Böttcher scores split by MA thresholds. Molecules from the COCONUT 2.0 database split into two groups based on a given MA threshold: **a,** MA=15, **b,** MA=16, **c,** MA=17, **d,** MA=18, **e,** MA=19, and **f,** MA=20. Then, the false-positive rate (FPR), false-negative rate (FNR), and overall accuracy were calculated regarding how well the Böttcher scores could split the data into the same two groups based on the corresponding Böttcher threshold for that given MA score (indicated by the vertical dotted black line). FPR values ranged from 0.19 to 0.32, FNR values ranged from 0.15 to 0.22, and accuracy values ranged from 0.76 to 0.81.

As Supplementary Fig. 2a shows, binary classification based on the COCONUT 2.0 molecules identified that a Bertz score of approximately 920.22 best separates molecules with an MA score of <15 from molecules with an MA score of ≥15. However, this process inflated the FPR from near-zero to 0.18, meaning we would witness an increase from 0% to 18% of non-biological molecules being falsely classified as biological molecules. Even if we select a more conservative non-life/life MA score cutoff threshold of 20 (which would presumably render the FPR from MA scores even closer to a value of zero), translation to Bertz scores would raise the FPR to a value of 0.21 (Supplementary Fig. 2f). While previous experimental evidence has suggested that using MA as a life detection tool would ensure negligibly small rates of false-positive declarations[15], an attempt to apply the same process via Bertz scores would likely create relatively common occurrences where molecules that come from non-life sources are falsely declared to provide evidence of life.

The situation is even more extreme when we investigate Böttcher scores[6]. Binary classification based on the COCONUT 2.0 molecules[16] identified that a Böttcher score of approximately 366.57 best separates molecules with an MA score of <15 from molecules with an MA score of ≥15 (Supplementary Fig. 3a). However, this process inflated the FPR from near-zero to 0.32, meaning we would witness an increase from 0% to 32% of non-biological molecules being falsely classified as biological molecules. Even if we again placed the MA score threshold to a more conservative value of 20 (which would create a FPR score even closer to zero), translation to Böttcher scores would elevate the FPR to a value of 0.19 (Supplementary Fig. 3f). While it would be ideal to provide a method that is conservative at labeling a molecule as providing evidence of life, we verify that this is likely better done using MA scores (as shown in previous studies), instead of using Böttcher (or Bertz) scores.

We derived a similar conclusion that MA scores cannot simply be translated into Bertz and Böttcher scores by plotting each score against one another. By examining this for all molecules in the COCONUT 2.0 database[16], we found that molecules with a Bertz score of approximately 1,000 translated into MA scores from as low as MA=6 (which would be well below the MA≈15 threshold for evidence of life) to as high as MA=25 (which would be well above the MA≈15 threshold for evidence of life) (see Fig. 3 from the main paper). A visual representation of the shortest pathway of construction for example molecules on these extreme ends are shown in Supplementary Figs. 4 and 5. The example MA=6 molecule (known as benzo[j]fluoranthene, B*j*F, which has been detected in meteorites)[17] requires only 2 building blocks and 6 construction steps (Supplementary Fig. 4), while the example MA=25 molecule requires 8 building blocks and 25 construction steps (Supplementary Fig. 5). Despite the clear difference in construction history difficulties (reflected in the clearly different MA scores), both molecules have similar Bertz scores of about 1,000.

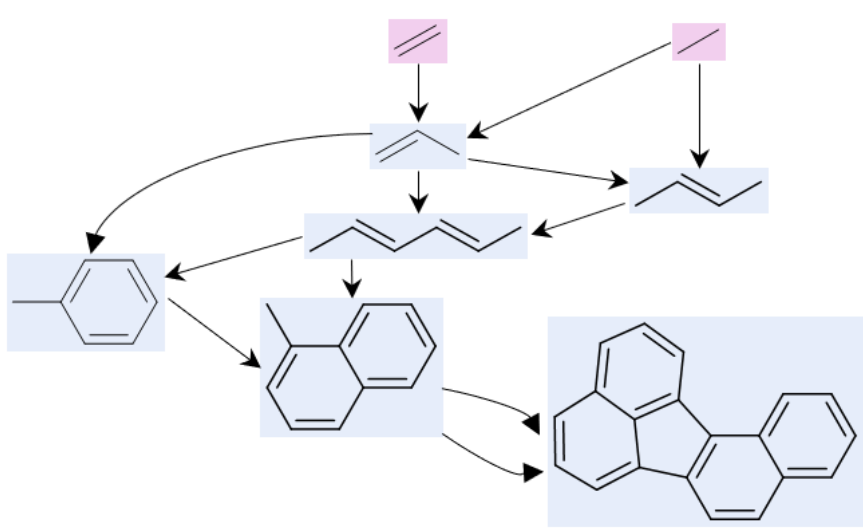

Supplementary Fig. 4: Shortest pathway of construction for example molecule #1 in Fig. 3b. This example molecule (known as benzo[j]fluoranthene, B*j*F, which has been detected in meteorites) with a Bertz score of approximately 1,000 can be formed in only six joining operations, giving it an MA score of six. This can be contrasted to another molecule with the same Bertz score but a much longer shortest pathway, as shown in Supplementary Fig. 4.

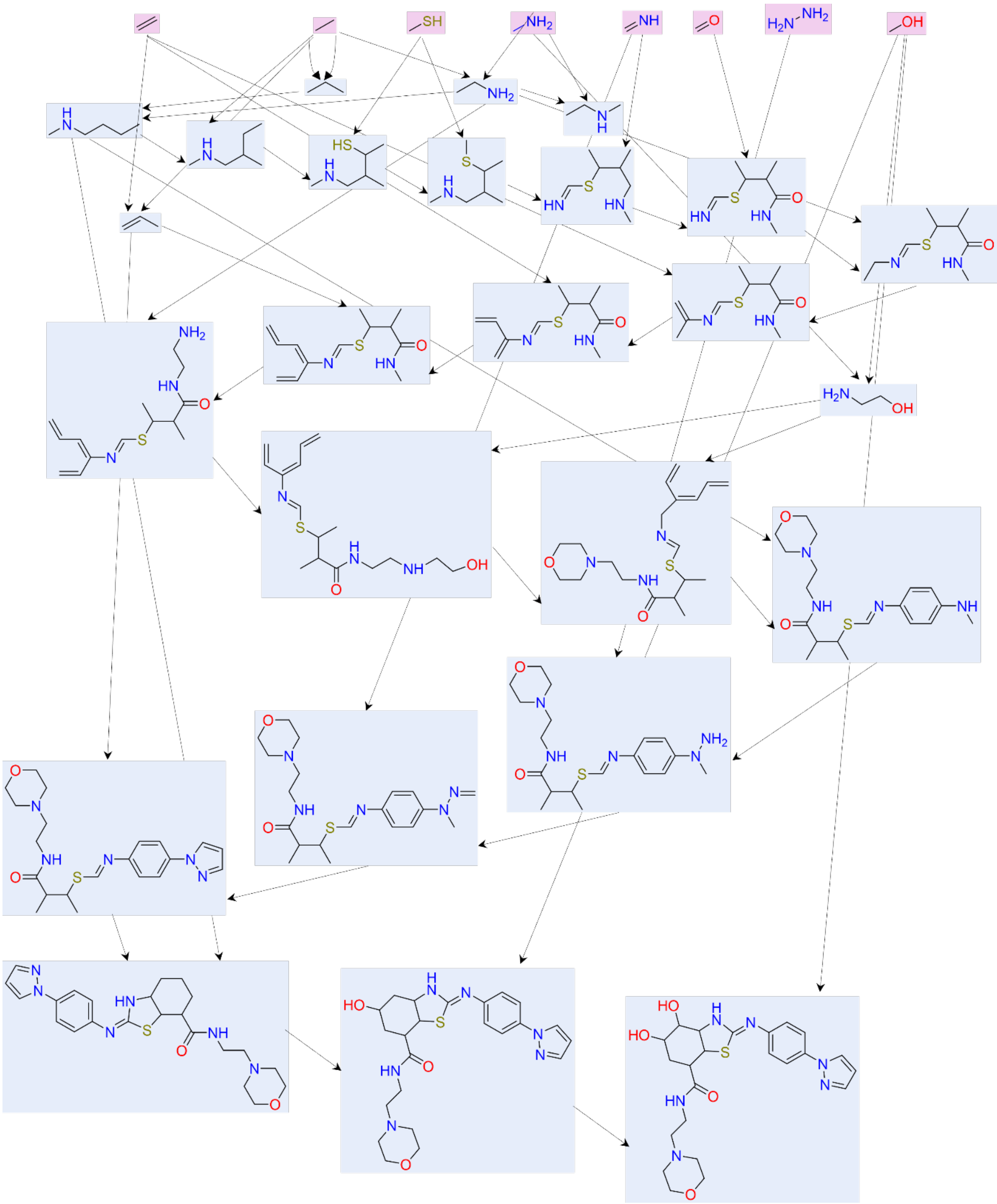

Supplementary Fig. 5: Shortest pathway of construction for example molecule #3 in Fig. 3b. This example molecule with a Bertz score of approximately 1,000 requires a minimum of 25 joining operations for its construction, giving it an MA score of 25. This can be contrasted to another molecule with the same Bertz score but a much shorter shortest pathway, as shown in Supplementary Fig. 4.

We explore additional comparisons between complexity metric scores plotted against one another in Supplementary Figs. 6-10. As a complement to Fig. 3 in the main paper, Supplementary Fig. 6 also compares the Bertz and MA scores for all molecules in the COCONUT 2.0 database; however, it now shows the spectrum of molecules with the same MA score of 10 but with vastly different Bertz scores (with a more than 26-fold variation). Molecule #1 contains 13 bonds and has a molecular weight of 189.26 Daltons (Da), while molecule #3 contains 72 bonds and has a molecular weight of 918.68 Da (Supplementary Fig. 6). Upon examining the structure of molecule #3, we observe that it contains repeating subunits that facilitate a shorter construction pathway, allowing it to maintain a lower MA score of 10 despite its large size (Supplementary Fig. 6). However, its Bertz score is notably high because the Bertz complexity metric does not detect these repeating motifs.

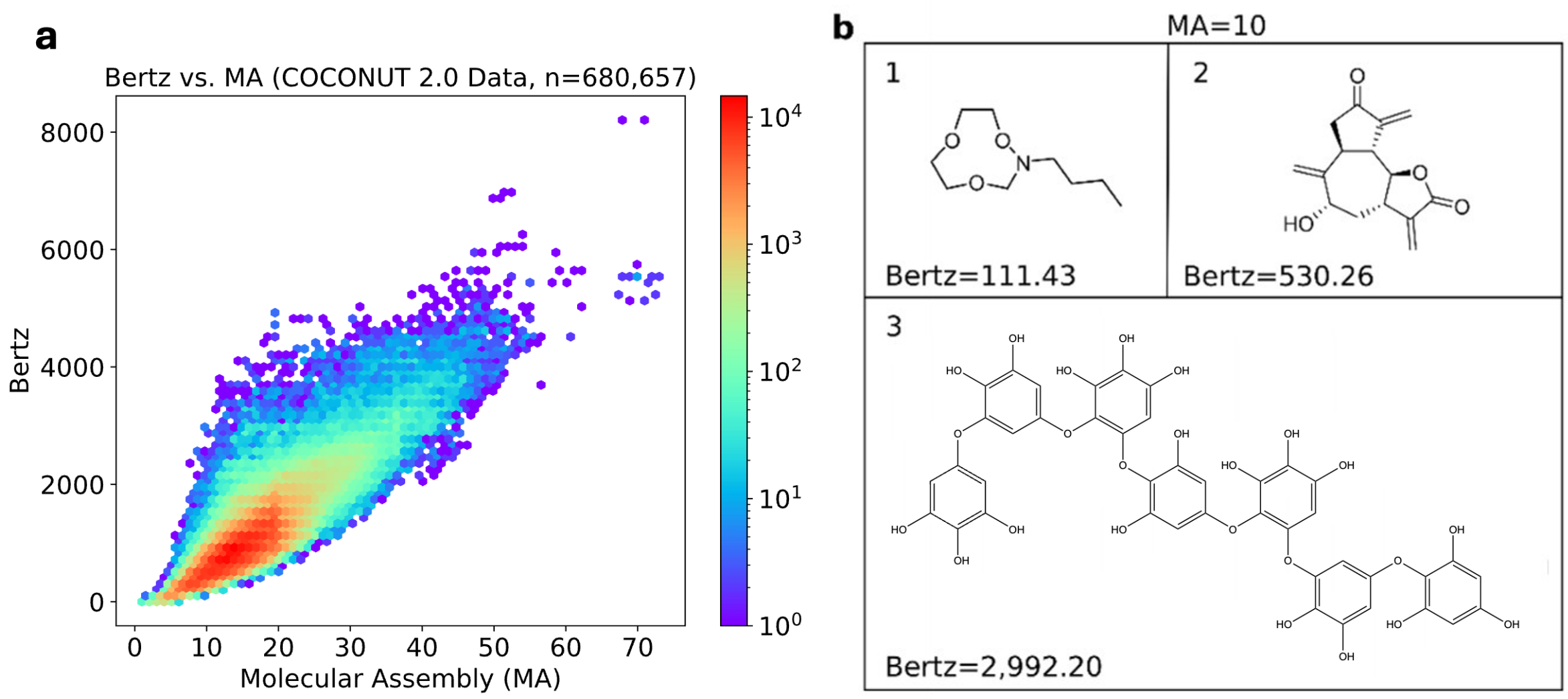

Supplementary Fig. 6: Comparing MA and Bertz scores, conditioned on constant MA scores. a, Bertz versus MA scores for each molecule in the COCONUT 2.0 database, with color indicating molecular count on a logarithmic scale for each hexagon bin. **b,** Three example molecules that each have the same MA score equal to 10, but with Bertz scores that are either low (111.427), intermediate (530.264), or high (2,922.203), demonstrating a more than 26-fold variation.

Having compared the Bertz and MA scores using this large-scale data-driven approach, we now perform the same analysis for the Böttcher and MA scores across all molecules in the COCONUT 2.0 database (Supplementary Fig. 7). Supplementary Fig. 7 shows the gamut of molecules with the same MA score of 10 but with vastly different Böttcher scores (with a more than 20-fold variation). Molecule #1 contains 17 bonds and has a molecular weight of 200.19 Da, while molecule #3 contains 78 bonds and has a molecular weight of 1,062.92 Da (Supplementary Fig. 7). Upon examining the structure of molecule #3, we observe that it contains multiple repeating subunits that facilitate a shorter construction pathway, resulting in a lower MA score relative to its large size (Supplementary Fig. 7). However, its Böttcher score is notably high because the Böttcher score does not as much consider these repeating motifs. Moreover, Böttcher scores increase as the number of stereocenter possibilities increases at each bond, rendering large molecules with many stereoisomers, such as molecule #3, as typically obtaining particularly high Böttcher scores (Supplementary Fig. 7).

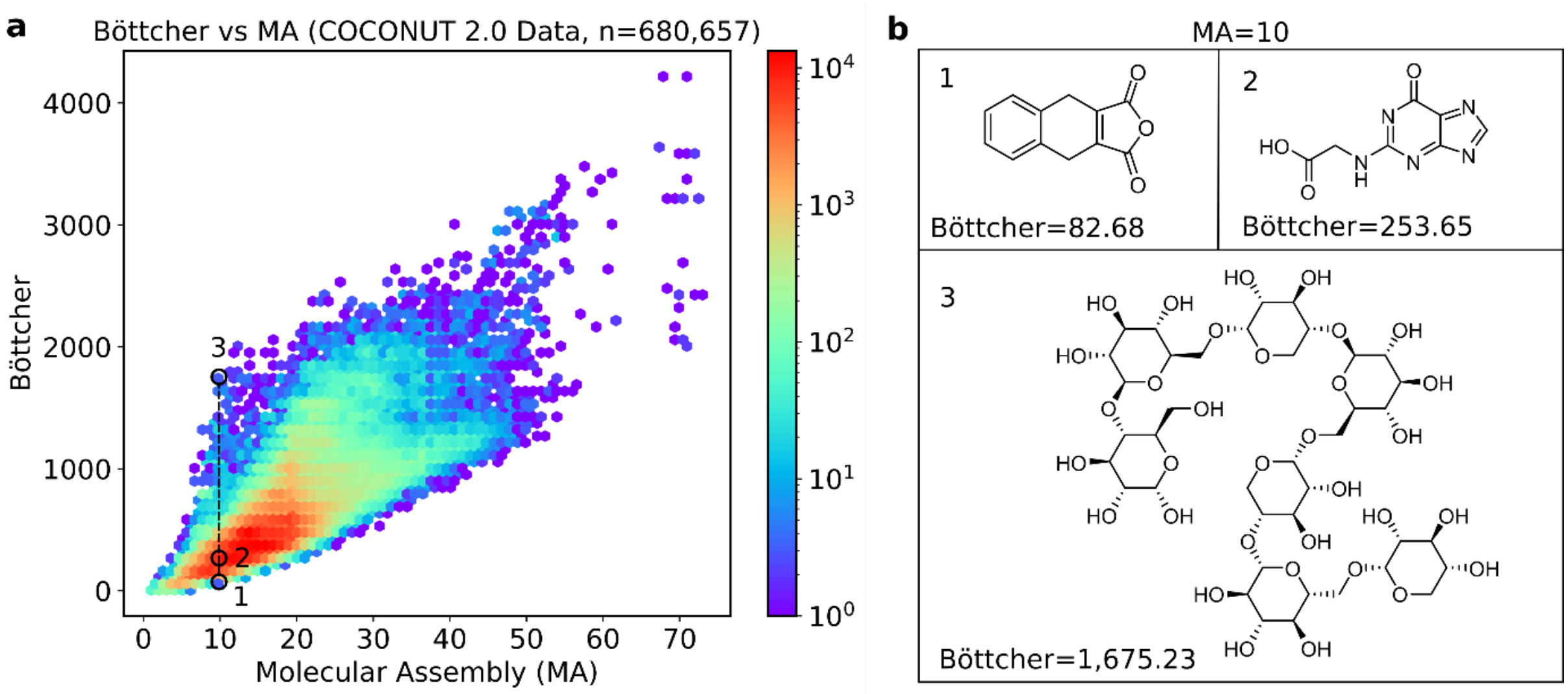

Supplementary Fig. 7: Comparing MA and Böttcher scores, conditioned on constant MA scores. **a,** Böttcher versus MA scores for each molecule in the COCONUT 2.0 database, with color indicating molecular count on a logarithmic scale for each hexagon bin. **b,** Three example molecules with a that each have the same MA score equal to 10, but with Böttcher scores that are either low (82.680), intermediate (253.648), or high (1,675.233), demonstrating a more than 20-fold variation.

Supplementary Fig. 8 shows the range of COCONUT 2.0 molecules with the same Böttcher score of about 500 but with vastly different MA scores (with a more than 4-fold difference). This means that Böttcher scoring criteria rates these molecules as having similar complexities, even though the shortest pathway of molecule #1 (which has 22 bonds and is 310.30 Da) can be accomplished with four-fold fewer joining operations than that of molecule #3 (which has 48 bonds and is 784.63 Da). Even though molecule #1 is smaller than molecule #3, it contains numerous stereocenters, which may increase its Böttcher score relative to its MA score (Supplementary Fig. 8). At the same time, molecule #1 has assembly pool objects that can be reused multiple times during its shortest pathway of construction, allowing it to possess a lower MA score, whereas molecule #3 is relatively heterogeneous and would require more assembly steps for its construction, allowing it to possess a higher MA score (Supplementary Fig. 8).

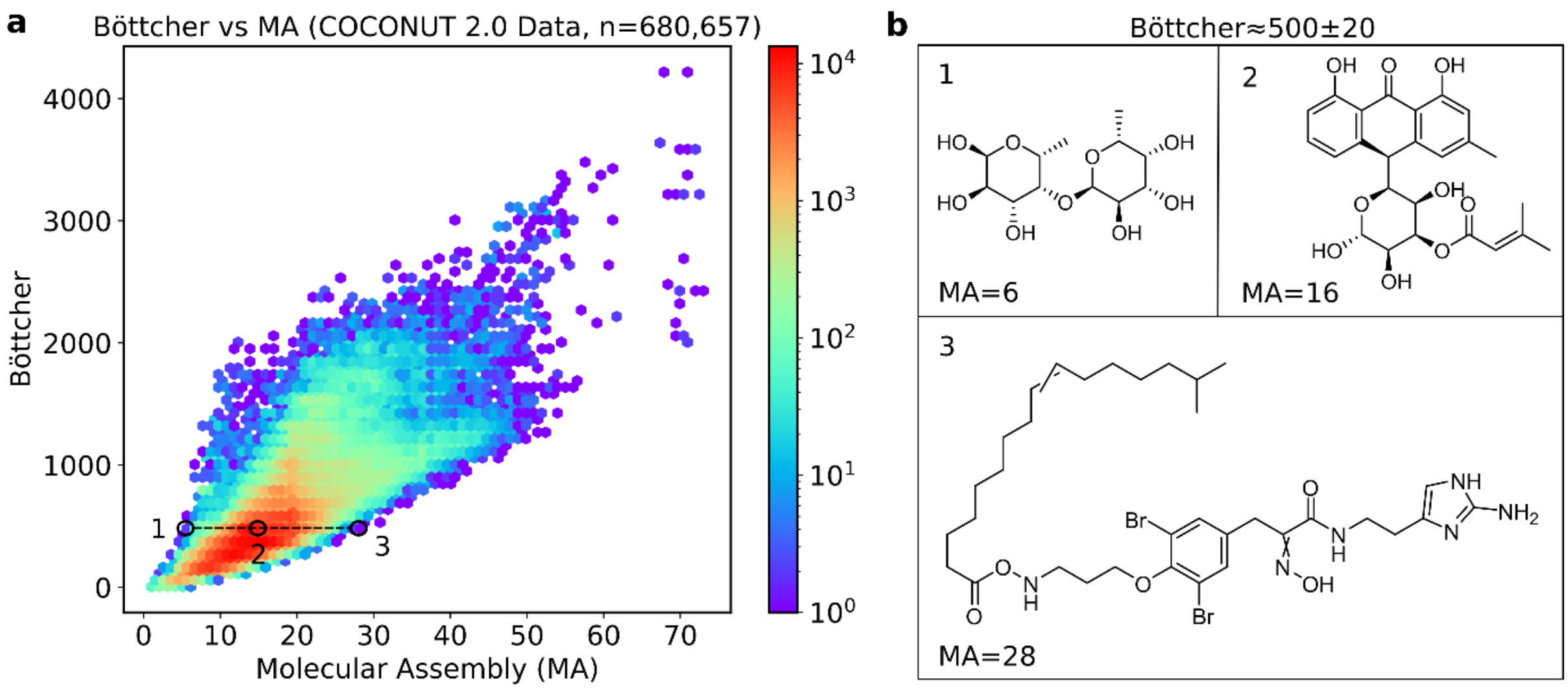

Supplementary Fig. 8: Comparing MA and Böttcher scores, conditioned on constant Böttcher scores. a, Böttcher versus MA scores for each molecule in the COCONUT 2.0 database, with color indicating molecular count on a logarithmic scale for each hexagon bin. **b,** Three example molecules that have the same Böttcher score of around 500, but with either a low (6), intermediate (16), or high (28) MA score, demonstrating a more than 4-fold difference.

Supplementary Figs. 9 and 10 show the range of COCONUT 2.0 molecules[16] when their Bertz and Böttcher scores are compared. We again see significant differences in relation to how the scores measure complexity, with molecules that possess topological complexity typically rating higher in Bertz scores and molecules that possess complexity in the atoms' microenvironments (such as stereocenters) typically rating higher in Böttcher scores (Supplementary Figs. 9 and 10).

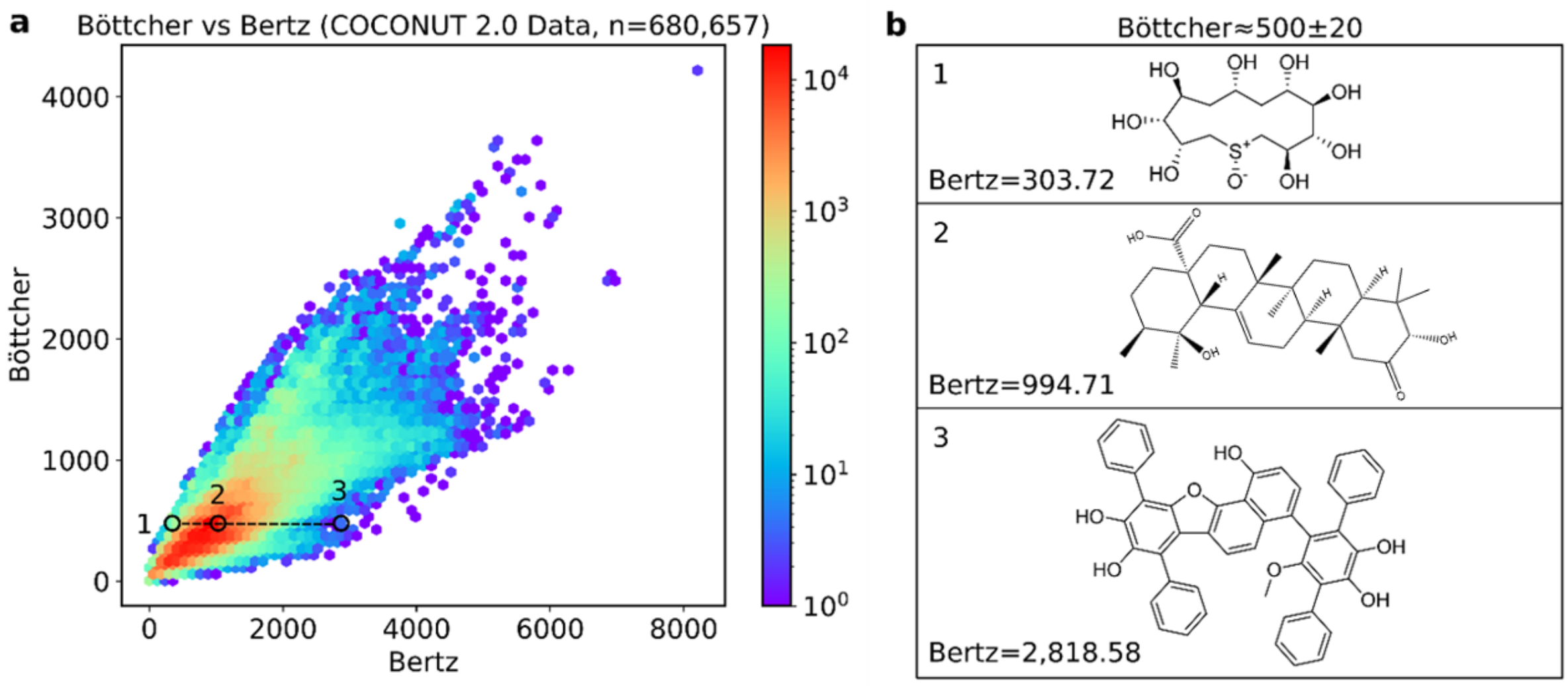

Supplementary Fig. 9: Comparing Böttcher and Bertz scores. a, Böttcher versus Bertz scores for each molecule in the COCONUT 2.0 database, with the color legend indicating the count of molecules on a logarithm scale. **b,** Three example molecules with similar Böttcher scores, but with either low (303.72), intermediate (994.71), or high (2,818.58) Bertz scores, demonstrating a more than 9-fold variation.

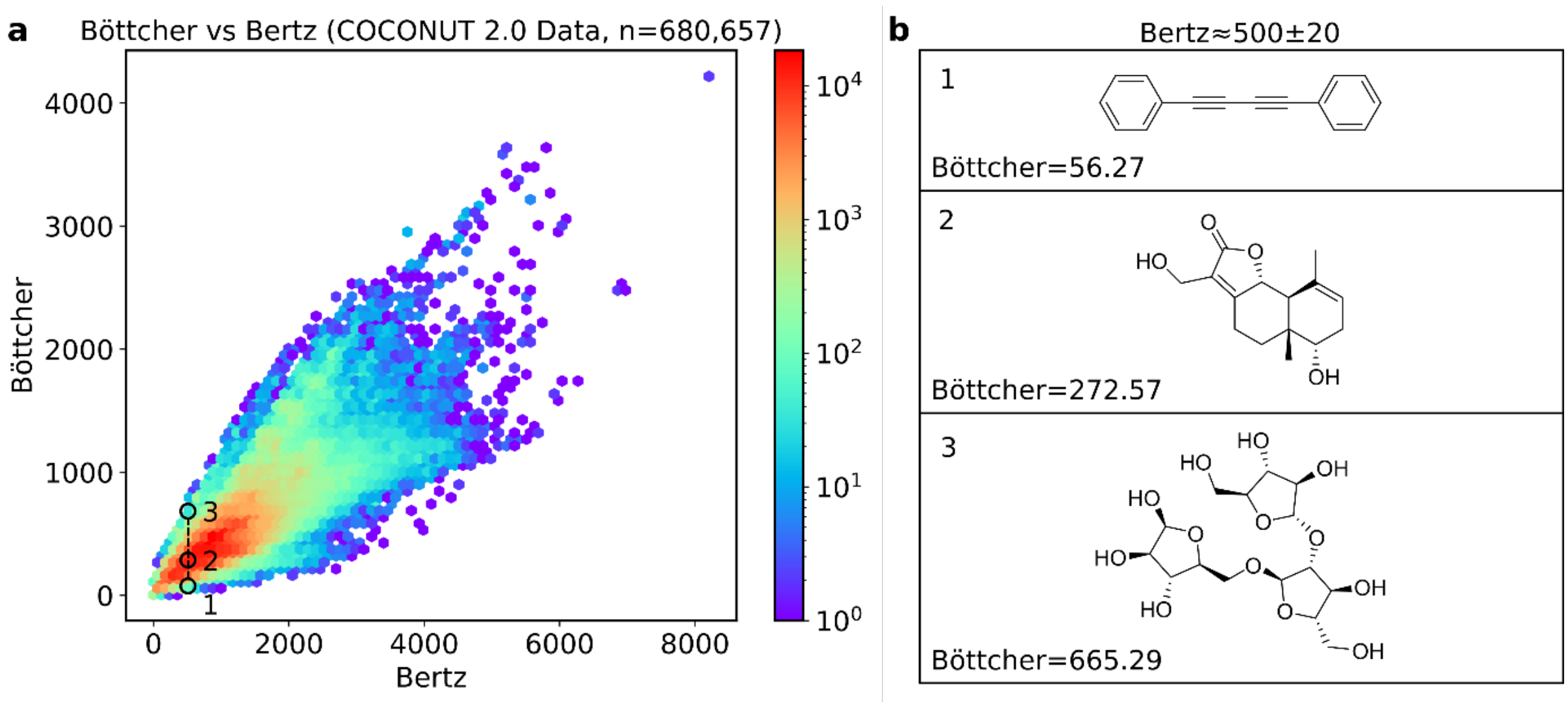

Supplementary Fig. 10: Comparing Böttcher and Bertz scores. a, Böttcher versus Bertz scores for each molecule in the COCONUT 2.0 database, with the color legend indicating the count of molecules on a logarithm scale. **b,** Three example molecules with similar Bertz scores, but with either low (56.27), intermediate (272.57), or high (665.29) Böttcher scores, demonstrating an almost 12-fold variation.

In Fig. 5 of the main paper, we compared complexity metrics across number of bonds (N_B) for the PubChem and COCONUT 2.0 databases[16,18], and reported differences between the complexity metrics. Supplementary Fig. 11 verifies that the patterns we observed are preserved even when we only consider the subset of molecules that contain no atoms other than carbon, hydrogen, nitrogen, and oxygen (CHNO molecules). This process reduced the full databases we saw in Fig. 5 to about 85% of COCONUT 2.0 and 40% of PubChem molecules.

Supplementary Fig. 12 verifies that the patterns we observed are preserved even when we only consider the inverse subset, i.e. molecules that contain at least one atom other than carbon, hydrogen, nitrogen, and oxygen (Non-CHNO molecules). This process reduced the full databases we saw in Fig. 5 to about 15% of COCONUT 2.0 and 60% of PubChem molecules. We see that the patterns persisted in both groups, suggesting that extended heteroatom diversity (i.e. inclusion of molecules with elements beyond CHNO) does not strongly influence the trends, although we do witness a slight decrease with how MA scores differentiate between

PubChem and COCONUT 2.0 molecules in the Non-CHNO subgroup (Supplementary Fig. 12).

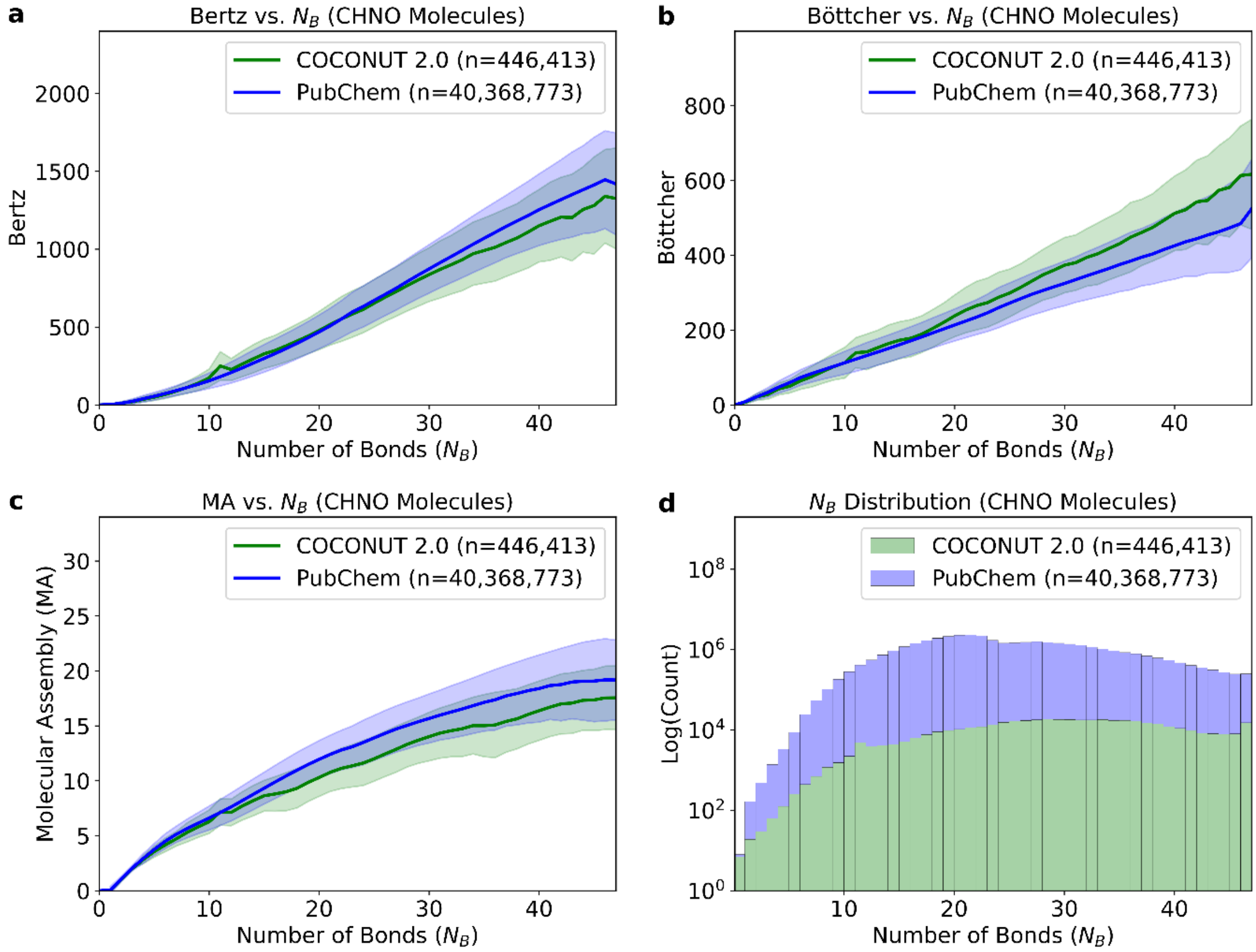

Supplementary Fig. 11: Molecular complexity growth rate per bond count for PubChem and COCONUT 2.0 CHNO molecules. Mean (solid line) and standard deviation (shaded area) of complexity scores per bond count between PubChem in blue (n ≈ 40,000,000) and COCONUT 2.0 in green (n ≈ 450,000) molecules that contain only carbon, hydrogen, nitrogen, and oxygen (CHNO). **a,** Bertz, **b,** Böttcher, and **c,** MA. **d,** The databases had similar distribution of bonds.

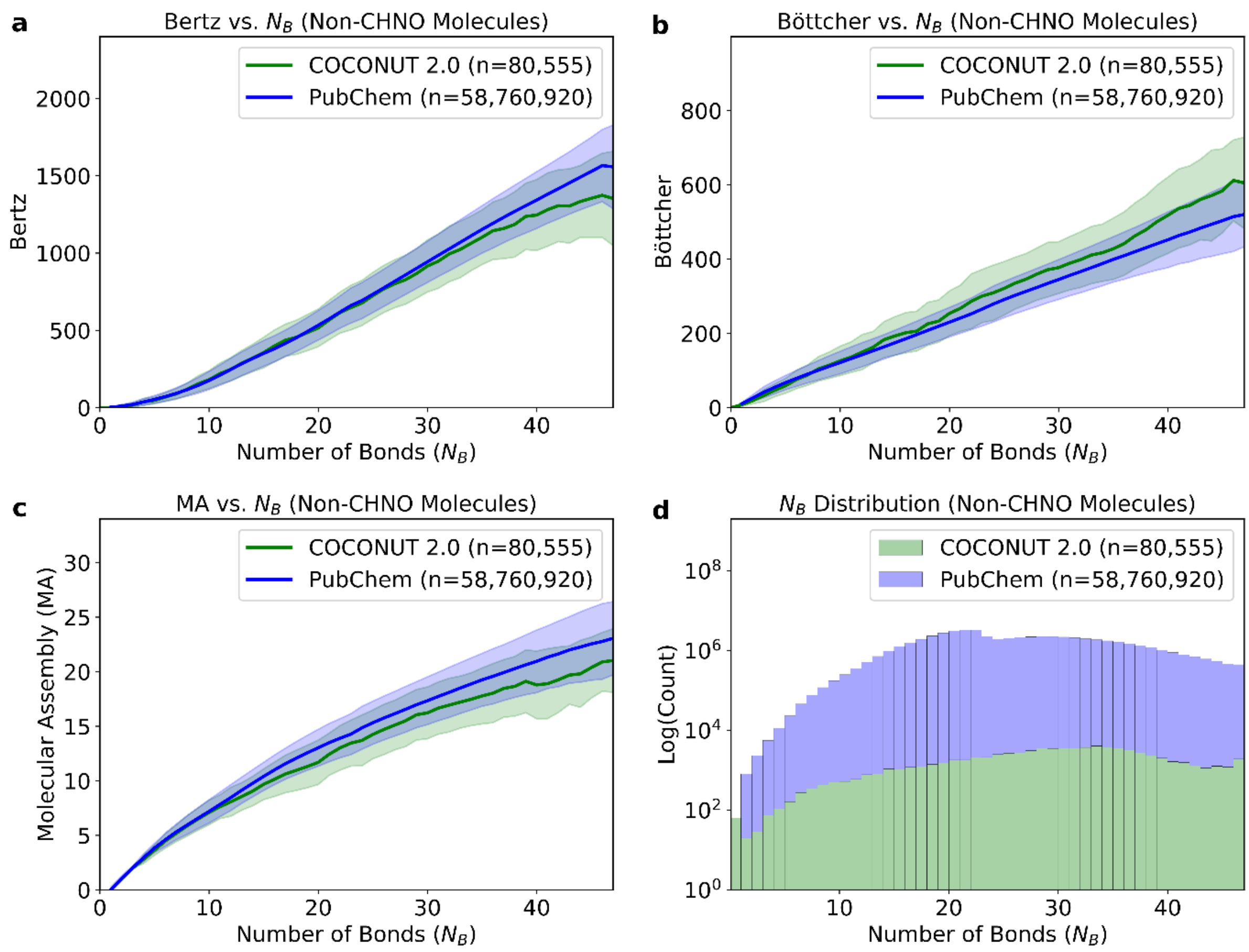

Supplementary Fig. 12: Molecular complexity growth rate per bond count for PubChem and COCONUT 2.0 Non-CHNO molecules. Mean (solid line) and standard deviation (shaded area) of complexity scores per bond count between PubChem in blue (n ≈ 40,000,000) and COCONUT 2.0 in green (n ≈ 450,000) molecules that contain at least one element besides carbon, hydrogen, nitrogen, and oxygen (Non-CHNO). **a,** Bertz, **b,** Böttcher, and **c,** MA. **d,** The databases had similar distribution of bonds.

We also examined the upper-bound of MA scores across both databases, and found that, for each N_B value, the highest MA score from PubChem was greater than or equal to the highest MA score from COCONUT 2.0 (Supplementary Fig. 13). The largest N_B value that maintained the strict upper-bound of $MA = N_B - 1$ occurred for $N_B = 14$ in the COCONUT 2.0 database, and occurred much later, at $N_B = 24$, in the PubChem database (molecules #1 and #2 in Supplementary Fig. 13). At $N_B = 43$, we observed the largest difference (8 MA units) between the maximum MA score from the PubChem versus that from the COCONUT 2.0 database. Upon inspecting these unusually heterogenous molecules, we found that PubChem contains

pharmaceutical compounds that require synthetic intervention, thus allowing for complex molecules to occur, even at relatively smaller molecular sizes. In contrast, COCONUT 2.0 molecules are molecules derived from natural products without any synthetic intervention. Overall, the PubChem molecules that contain high MA scores (especially above any N_B-matched COCONUT 2.0 molecules) could potentially be investigated as "molecular technosignatures", i.e. compounds that are unlikely to occur naturally without technological intervention[19,20] (Supplementary Fig. 13).

Example molecules with unusually high MAs in the PubChem database are shown in Supplementary Fig. 13. These example molecules reveal molecular motifs that are likely to add to their high MAs relative to their sizes. For instance, the terminal positions of the molecules often contain trifluoromethyl groups (all four molecules), tert-butyl groups (molecules #2 and 4), and phosphate groups (molecule #1). The internal positions of the molecules contain motifs such as a mixed boronate anhydride (molecule #1), silyl ethers (molecule #2 and 4), sulfonyl groups (all molecules), and heterocyclic rings (molecules #1, 2, and 4). Methylene linkers and other structural linkers are also present in the molecules, bridging complex functional groups and allowing the molecules to grow in size and structural diversity. Interestingly, several of these motifs are unlikely to occur in natural products: for example, trifluoromethyl groups[21,22], organoboron compounds[23], and silyl ethers are found almost exclusively in synthetic compounds, while tert-butyl groups and sulfonyl groups are also usually synthetic.

We also examined the lower-bound of MA scores across both databases, and found that, for each N_B value, the lowest MA score from PubChem was less than or equal to the lowest MA score from COCONUT 2.0 (Supplementary Fig. 13). This is likely because PubChem contains mineral structures, which have structural repetitions that translate into low MAs.

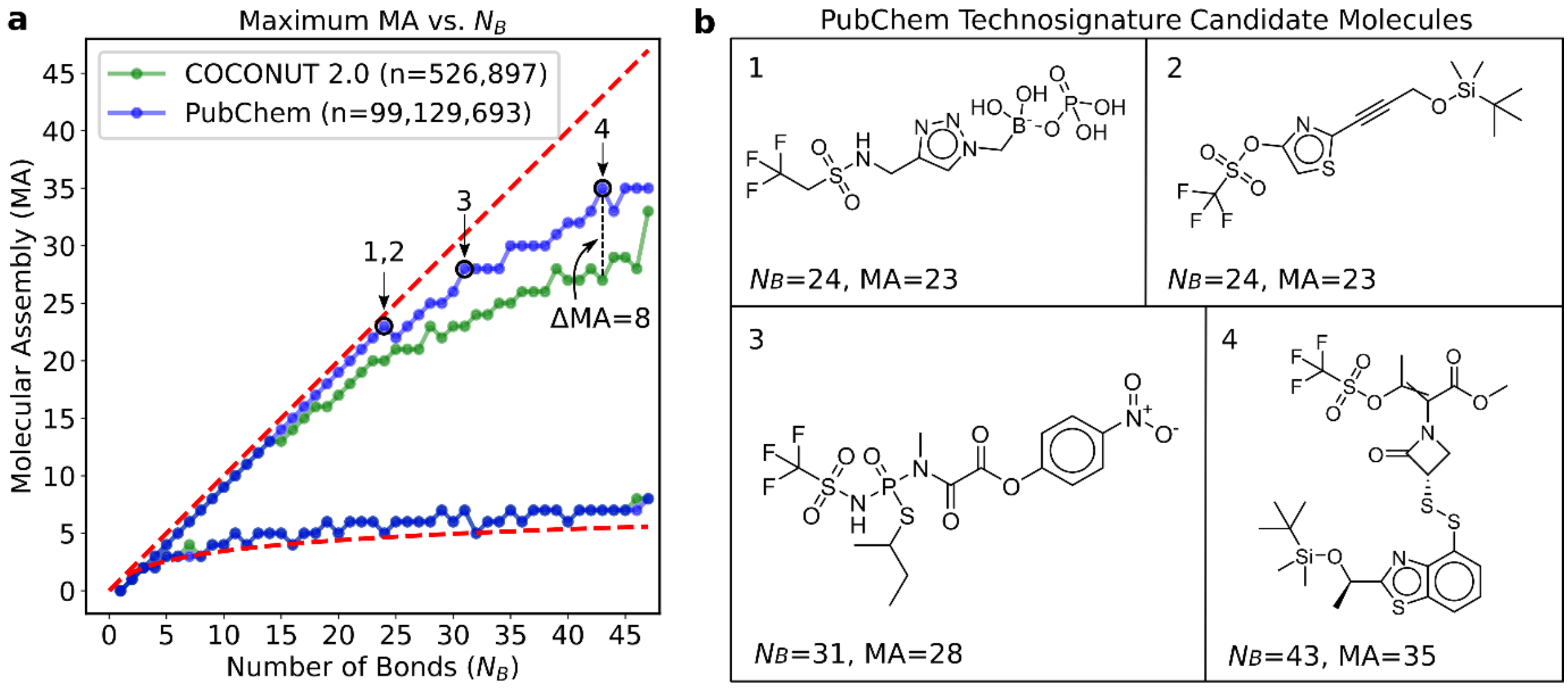

Supplementary Fig. 13: Highest MA scores per number of bonds. The two dotted red lines demonstrate mathematically derivable bounds of MA scores, with an upper bound of $MA = N_B - 1$ and a lower bound of $MA = log_2(N_B)$, where N_B represents the number of bonds. The highest MA score in PubChem was greater than or equal to that of COCONUT 2.0 per N_B. The largest molecule to maintain $MA = N_B - 1$ in the COCONUT 2.0 database occurred at $N_B = 14$, whereas the largest molecule to maintain $MA = N_B - 1$ in the PubChem database occurred much later, at $N_B = 24$. Molecules 1 and 3 have 13 building blocks, while molecules 2 and 4 have 12 building blocks.

3. Evaluating ML prediction of MA from MS[1] data

As reported in the main paper, we found that our XGBoost model reduced the relative MSE error by approximately three-fold compared to baseline models regressed on molecular weight (Supplementary Table 2). We also found that our XGBoost model appeared to learn efficiently, based on the visual diagnostics of its learning curve (Supplementary Fig. 14). Specifically, the training loss consistently decreases and so does the testing loss (Supplementary Fig. 14). There is a minimal gap between the training and testing error, and they both plateau after many boosting rounds (Supplementary Fig. 14). The curves are also smooth. These visual diagnostics altogether suggested that the model is learning sufficiently, is not over-fitting or under-fitting, and would likely generalize well to similar databases (Supplementary Fig. 14). We indeed

confirmed that our model generalized well to other databases: for example, when we applied our XGBoost model (that was trained on the NIST SRD database) to the MassBank database (even without any updates to the optimal hyperparameters), the relative MSE increased (from 0.04 to 0.07) but was still less than the best baseline models trained and tested on the NIST SRD database (Supplementary Table 2). Sample molecules where the XGBoost model showed improvements over baseline models are noted to highlight its performance (Supplementary Table 3). The results for the XGBoost models on simulated MS data are discussed in the main paper (Supplementary Table 4).

	Linear	Log	Power	Polynomial	ML NIST	ML MassBank
Relative MSE	0.15	0.13	0.12	0.12	0.04	0.07

Supplementary Table 2: MA predictability from simple models and XGBoost. Relative MSE of baseline models (linear, logarithmic, power law, and second-degree polynomial) for MA regressed on MW, plus relative MSE of our XGBoost model tested on the training subset of the NIST SRD database[1] and relative MSE of our XGBoost model tested on the MassBank. All cases used all molecules with mass spectra available on the NIST SRD database[1].

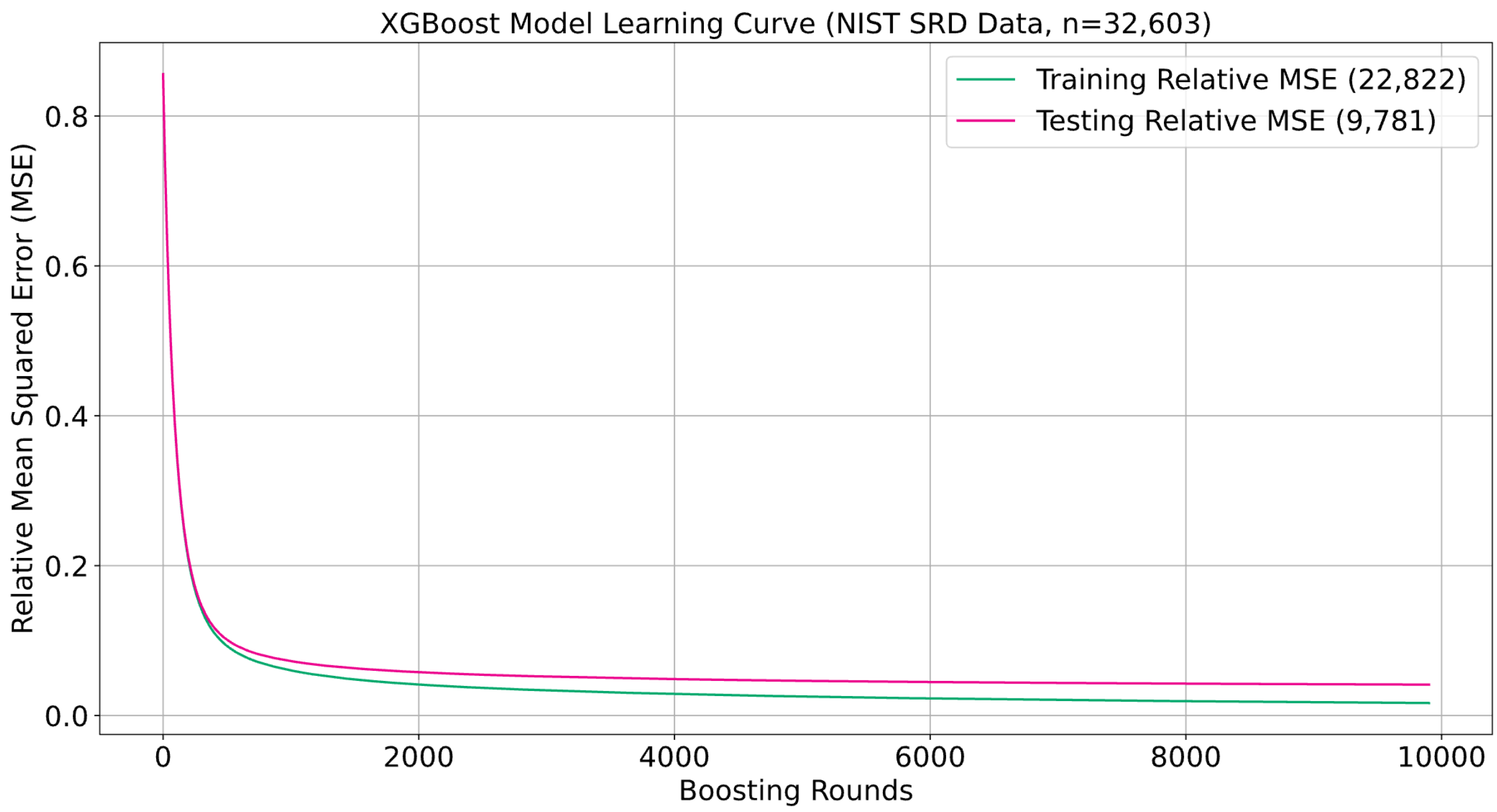

Supplementary Fig. 14: Learning curve of the XGBoost model. Learning curve of training and testing data in the XGBoost model applied to the NIST SRD database, with the x-axis showing boosting rounds and the y-axis showing relative MSE. The learning curve confirms that the model is likely performing well.

Molecule		y	$\hat{y}_{xgb}$	$\hat{y}_{bpl}$	Δ	FP/FN
a	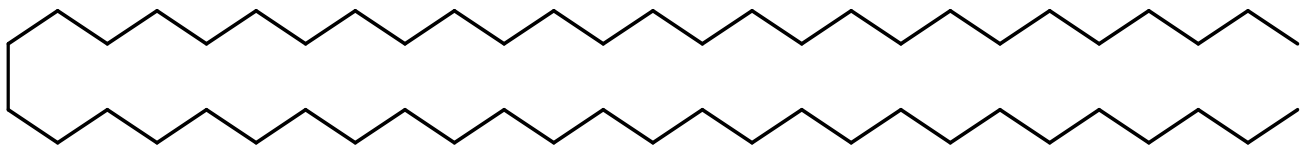	8	7.9	21.1	13.1	+
b	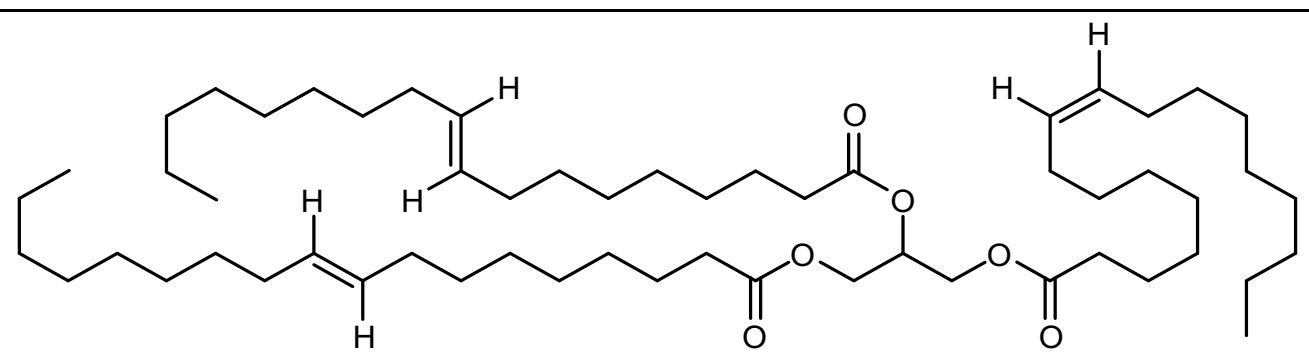	11	10.8	23.8	12.6	+
c	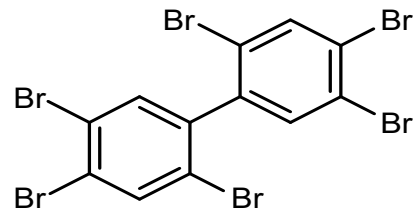	7	7.8	18.1	10.3	+
d	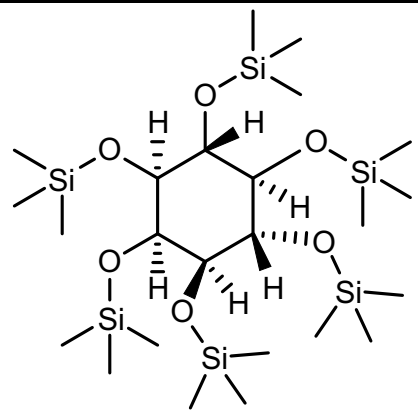	8	8.2	17.8	9.6	+
e	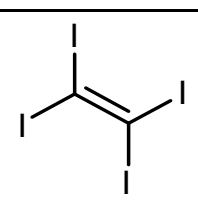	3	3.1	15.9	12.9	+
f	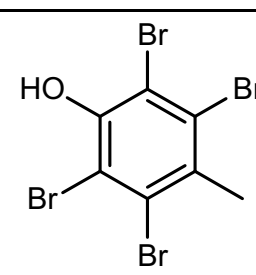	7	6.8	13.3	6.1	
g	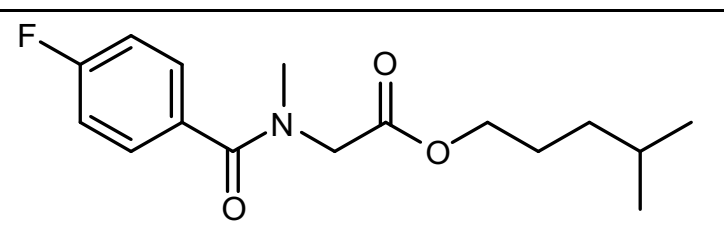	16	15.4	10.0	5.3	−

Supplementary Table 3: Example molecules with improved MA predictions using XGBoost over baseline models. The true MA value (y), the XGBoost-predicted MA value ($\hat{y}_{xgb}$), the baseline power law predicted MA value ($\hat{y}_{bpl}$), the reduction in error when predicting with XGBoost instead of the baseline model (Δ, calculated as $\Delta = |y - \hat{y}_{bpl}| - |y - \hat{y}_{xgb}|$), and whether the baseline model would result in a false positive or false negative result (FP/FN), where a FP (+) result occurs when $y < 15 \cap \hat{y}_{bpl} \geq 15$, and a FN (−) result occurs when $y \geq 15 \cap \hat{y}_{bpl} < 15$.

Train / Test	10eV-only	20eV-only	40eV-only	Split-eV	Integrated-eV
10eV-only	0.034	0.036	0.041	0.037	0.037
20eV-only	0.050	0.031	0.036	0.040	0.031
40eV-only	0.061	0.044	0.031	0.045	0.035
Split-eV	0.038	0.033	0.033	0.035	0.032
Integrated-eV	0.067	0.040	0.037	0.049	0.029

Supplementary Table 4: Relative MSE for XGBoost models on simulated MS data. XGBoost results for simulated MS data generated under five conditions: single-energy levels (10eV-only, 20eV-only, and 40eV-only), a mixed-energy level (1/3 of samples are each drawn from a different energy level), and an integrated-energy level (all samples combine spectra from all three energy levels) for all molecules on the NIST SRD database that had MS data. Cell values indicate relative MSE values, row names indicate the training dataset, and column names indicate the testing dataset.

4. Determining instrument effects on ML error using simulated MS

We realized that MS instrument parameters affected our model after we witnessed an approximately 1.75-fold increase in the relative MSE (from 0.04 to 0.07) when our XGBoost model (which was trained on the NIST SRD database) was tested on the MassBank database (Supplementary Table 2). In this section, we investigate how limitations in MS instrumentation affect the performance of our model. We primarily used the CFM-ID software, which allowed us to generate simulated MS data at different energy levels (10eV, 20eV, and 40eV) for each of the molecules in the original NIST SRD database (minus 14 molecules, for which the CFM-ID software could not produce simulated MS data). We vectorized the simulated CFM-ID MS data in the same manner as we did for the NIST SRD MS data, as described in Supplementary Materials Section 1.6. However, we note that the CFM-ID MS data did not only contain integer m/z values as was the case for the NIST SRD MS data. As a result, for the CFM-ID MS data, we rounded each peak intensity to the nearest whole value; in the small subset of cases for which two or more peaks rounded to the same nearest whole value, we kept the largest peak

intensity. Then, just as we did for the NIST SRD data, we normalized the vectorized MS data so that all peak intensity values were between zero and unity (Supplementary Materials Section 1.6).

First, we examined how energy levels affected MS (Supplementary Fig. 15). Energy levels slightly influenced the maximum peak m/z in a subset of samples. Namely, higher energy levels (40eV) sometimes fragmented the parent molecule, causing the m/z of the highest peak to be less than the molecular weight of the parent molecule (Supplementary Fig. 15b). This trend decreased as the energy levels lessened to 20eV (Supplementary Fig. 15c), while the 10eV MS rarely fragmented the parent molecule (Supplementary Fig. 15d). The trend was most apparent in the original NIST SRD data, which mostly used higher energy levels (70eV) and a harsher ionization technique (EI) than the simulated data (ESI) (Supplementary Fig. 15a). Despite these trends, most of the ~32,600 molecules maintained a maximum m/z value close to the molecular weight of the parent molecule, as most points in the scatterplots aligned to the x=y line (Supplementary Fig. 15).

Energy levels influenced the minimum peak m/z (Supplementary Fig. 16a) even more strongly than the maximum peak m/z, which did not display as much of a prominent difference when visualized through side-by-side boxplots (Supplementary Fig. 16b). Smaller energy levels resulted in the widest range of minimum peak m/z values (Supplementary Fig. 16a). Higher energy levels tended to result in a slightly larger number of peaks (Supplementary Fig. 16c) and moderately larger intensities across those peaks (Supplementary Fig. 16d). Some of these trends are verified in Fig. 6b with the example molecule phenylalanine (which has been identified in the ISM and meteorites). In this case, higher energy levels tended to capture smaller fragments with peaks of higher intensities, whereas lower energy levels tended to

capture larger fragments with peaks of smaller intensities (Fig. 6b and Supplementary Fig. 17). Supplementary Fig. 17c shows the detailed annotation of each molecular fragment in the simulated MS datasets; this demonstrates the physical and conceptual relationship between MS data and MA score calculation from the molecular fragmentation tree.

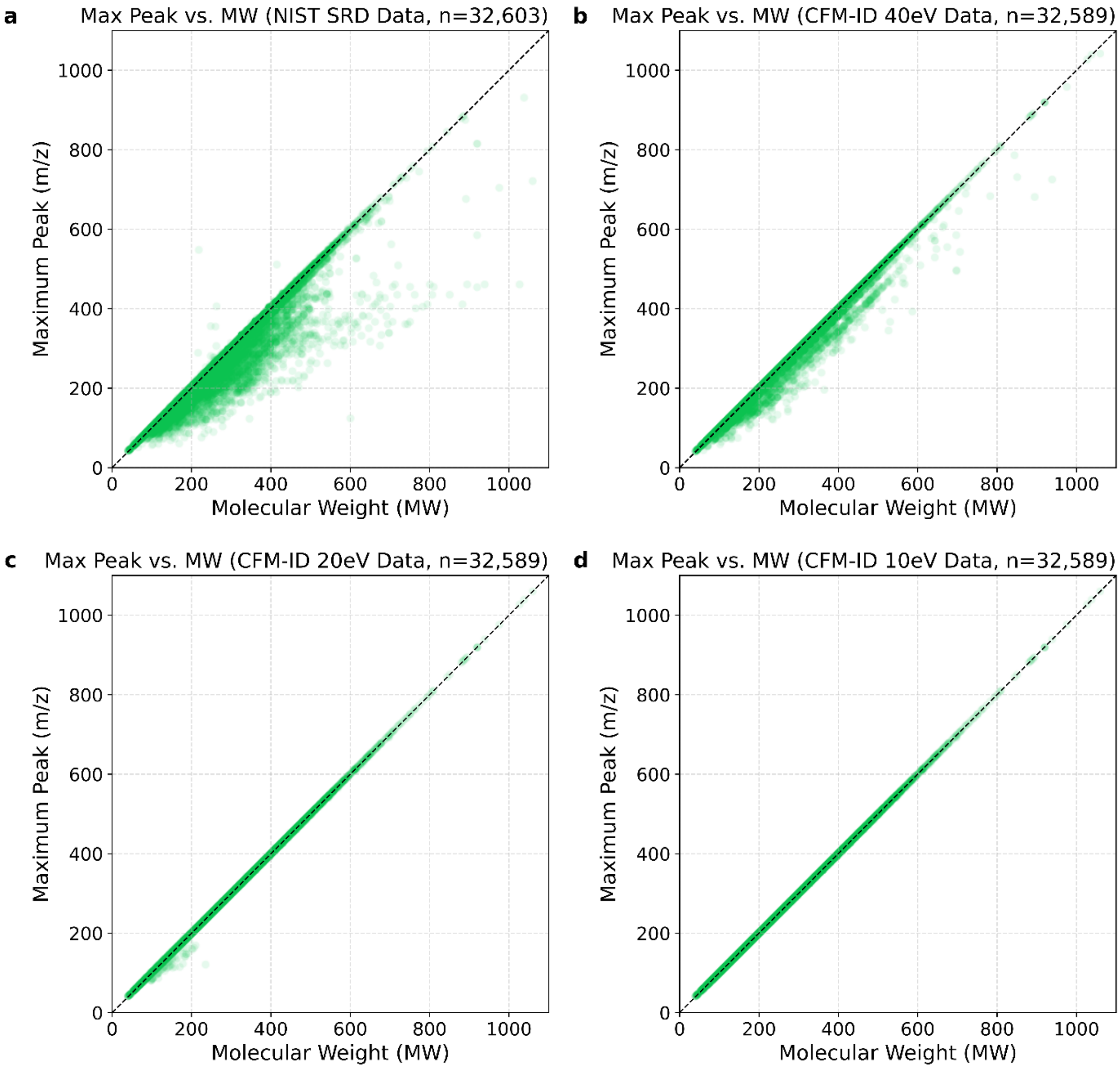

Supplementary Fig. 15: Largest peak m/z versus molecular weight. The m/z value of the largest peak is not always equal to the molecular weight of the parent molecule. **a,** The NIST EI data (which is mostly 70eV) showed the largest number of molecules for which the largest m/z value was less than the molecular weight. **b-d,** The CFM-ID ESI data showed the trend less prominently, especially as the energy levels decreased from 40eV (**b**) to 20eV (**c**) to 10eV (**d**).

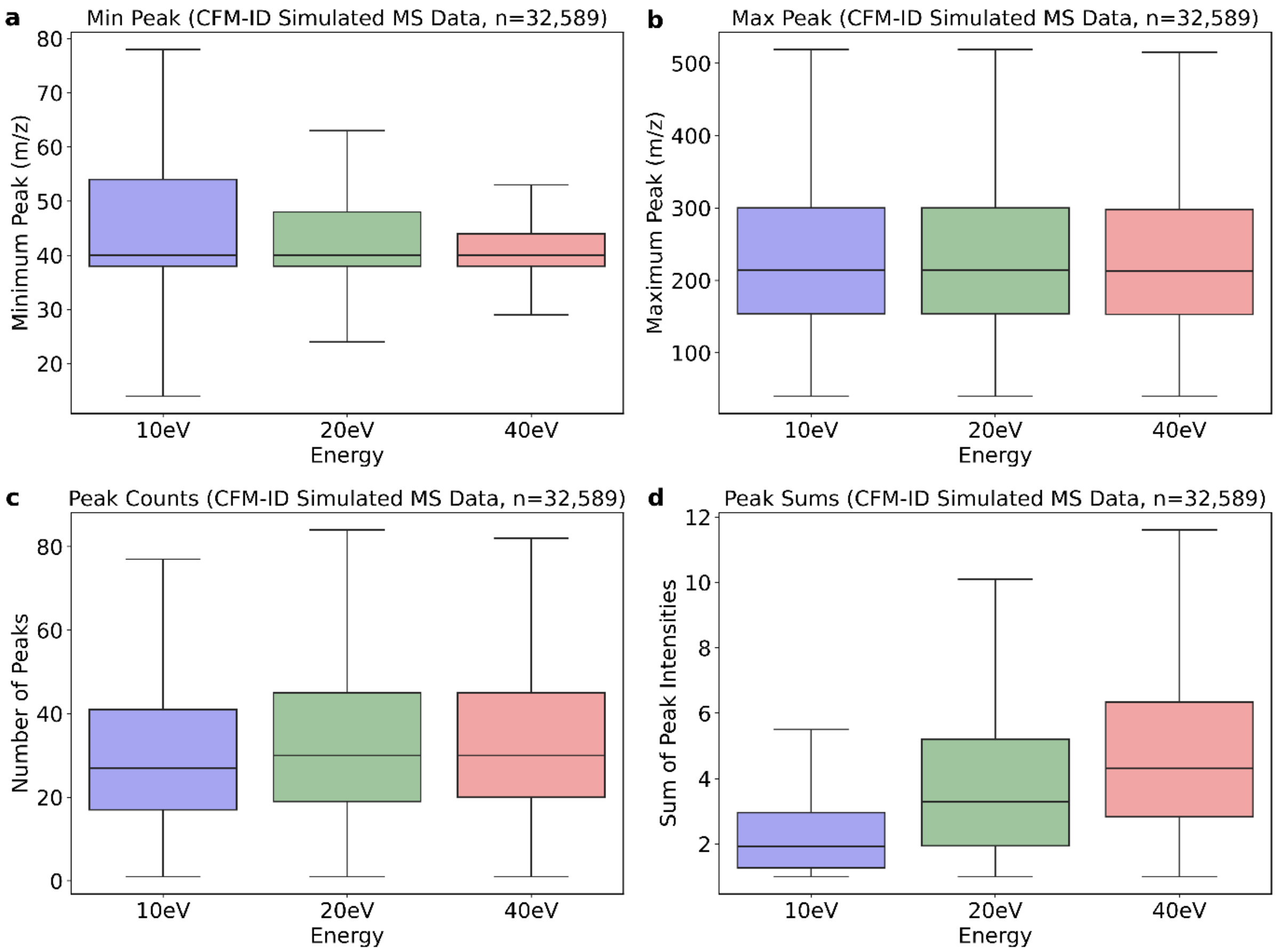

Supplementary Fig. 16: MS differences in simulated data at different energy levels. a, Smaller energy levels produced wider variance in the m/z value of the smallest peak for the same set of molecules. **b,** Different energy levels do not produce noticeable differences in the m/z value of the largest peak for the same set of molecules when viewed as side-by-side boxplots. However, as shown in Supplementary Fig. 15, higher energy levels do contain a larger subset of molecules that have m/z values in their largest peaks that are smaller than the molecular weight of the parent molecule. **c,** Higher energy levels tend to produce a larger number of peaks in the MS data. **d,** Higher energy levels tend to produce higher peak intensities in the MS data.

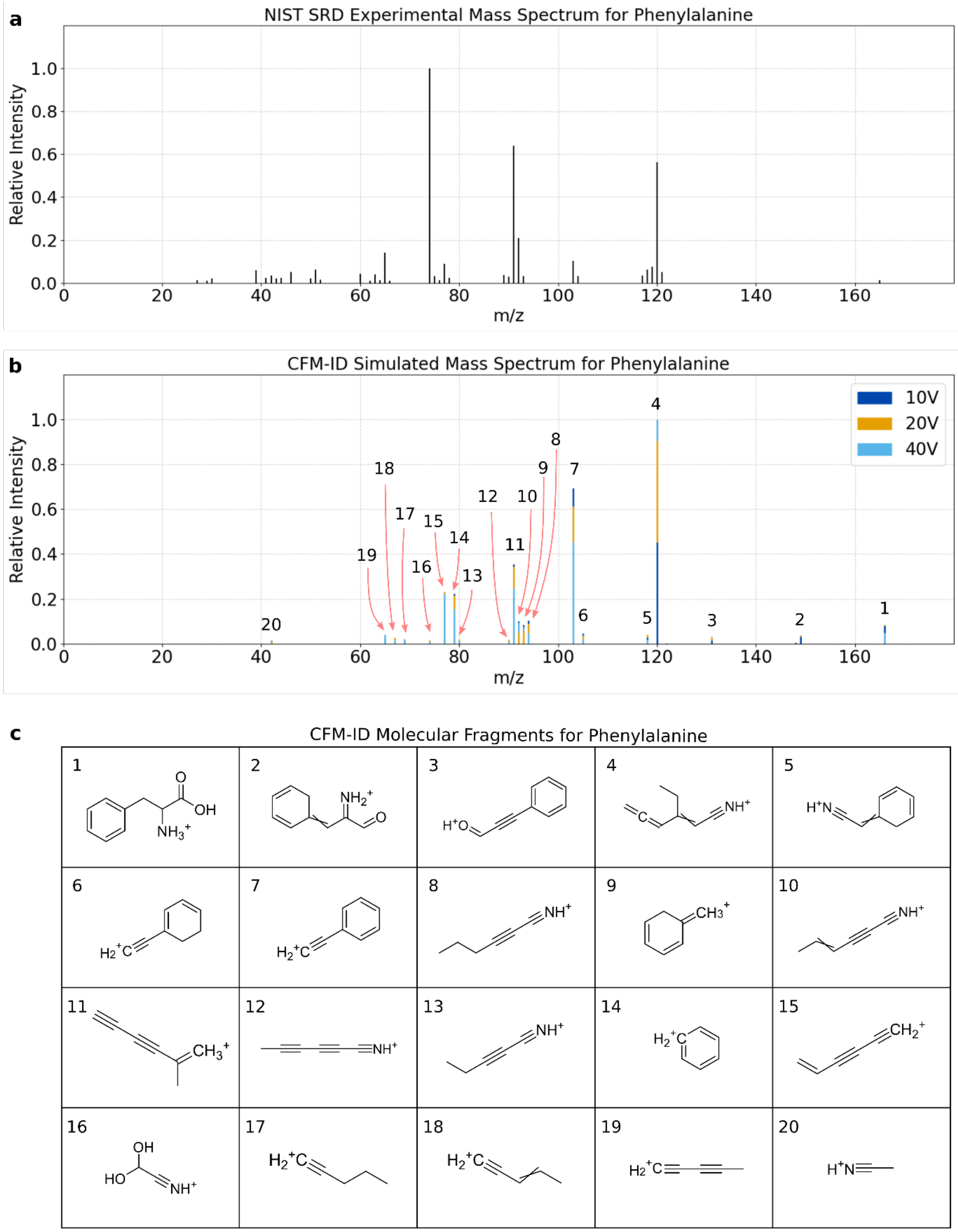

Supplementary Fig. 17: Example fragment identification in MS for phenylalanine. a, Experimental MS of phenylalanine from the NIST SRD database. **b,** Simulated MS of phenylalanine from the CFM-ID software run at three energy levels. Peaks from the MS of each energy level were concatenated together and normalized between 0 and 1; the height of colors in each peak represents the contribution from the corresponding colors in the legend. Peaks with more than one color are arranged with the largest contributor (highest peak intensity) at the bottom. Higher energy levels tended to contribute to smaller m/z fragments at high intensities. **c,** The CFM-ID software includes fragment identification.

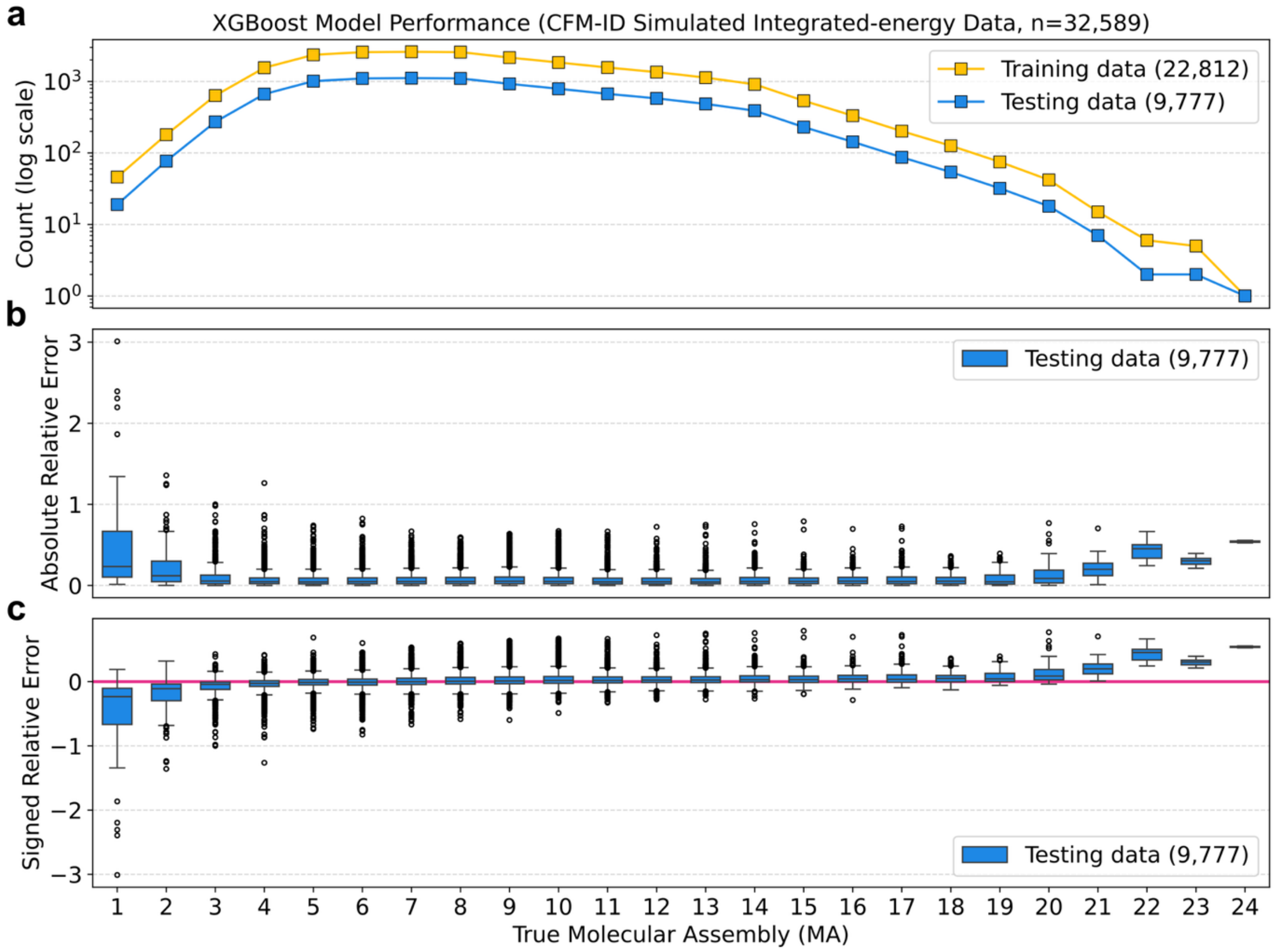

Supplementary Fig. 18: XGBoost model performance on the CFM-ID simulated integrated-energy data. a, Count of molecules per MA bin for the training and testing data, **b,** absolute relative error (calculated as $|y - \hat{y}|/y$), and **c,** signed relative error (calculated as $(y - \hat{y})/y$)), where y equals the true MA score and $\hat{y}$ equals the predicted MA score. Comparing **a** and **b**, we see an anti-correlation between the absolute relative errors and the count of molecules for each MA score in the data. As **c** shows, low MA molecules tended to be over-predicted (leading to a negative signed prediction error) and high MA molecules tended to be under-predicted (leading to a positive signed prediction error). This trend revealed that our model was conservative in mitigating false positive assignment of life detection (i.e. was unlikely to overestimate the MA scores of high MA molecules).

We trained and tested XGBoost models on each single-energy level (10eV-only, 20eV-only, and 40eV-only), a mixed-energy level (1/3 of samples are each drawn from a different energy level), and an integrated-energy level (all samples combine spectra from all three energy levels) (Table 4). Of these models, we expected the integrated-energy level to perform the best as it simultaneously contained information across all three energy levels, allowing it to cover a bigger range of the different types of molecular fragments (Supplementary Figs. 15-17 and Fig.

6b). We then verified that indeed the integrated-energy level performed the best, resulting in the smallest relative MSE (0.029) (Table 4, Supplementary Fig. 18).

While all our XGBoost models (including our single-energy simulated CFM-ID models) performed much better than baseline models, we still wished to determine what types of molecules were less accurately predicted (i.e. contributed most to the error in our models). Using the 40eV-only data, we discovered a clear relationship between the number of MS peaks and error (Supplementary Figs. 19, 20). In general, molecules that were significantly underpredicted tended to have a smaller number of peaks compared to other molecules with the same MA score, and molecules that were significantly overpredicted tended to have a larger number of peaks compared to other molecules with the same MA score (Supplementary Figs. 19, 20). In some cases, these outliers had relatively larger m/z values in their maximum peak compared to other molecules with the same MA score, suggesting that they tend to be heavier molecules relative to their MA scores.

Altogether, this post-hoc analysis suggests a trend that heavier molecules that break into an unusually large number of fragments can increase the chance of an overprediction, while heavier molecules that do not break at all or only break into an unusually small number of fragments can increase the chance of an underprediction in our XGBoost model. Supplementary Fig. 21 illustrates this pattern with example molecules that were strongly overpredicted and strongly underpredicted outliers. The overpredicted molecules tended to contain long hydrocarbon chains that are easy for the instrument (even in softer ESI techniques) to break apart into many small fragments (Supplementary Fig. 21a, c). We note that these same types of molecules tend to be even more extensively overpredicted in baseline models (Table

3a,b), allowing for our model to usually still outperform in these cases by overpredicting their MA scores to a lesser extent.

The underpredicted molecules were types that the instrument does not tend to fragment easily (especially in softer ESI techniques), such as fused benzene rings and benzene rings with steric hindrance caused by halogens (Supplementary Fig. 21b, d). In fact, some of these molecules that were unusually underpredicted in our model simply flew through the MS instrument without breaking into any fragments at all, resulting in only a single peak (Supplementary Fig. 21b, d). For the 925 molecules in the dataset with MA=9, a median of more than 30 peaks were generated in the MS (Supplementary Fig. 19i), and for the 390 molecules in our dataset with MA=14, a median of more than 50 peaks were generated in the MS (Supplementary Fig. 20i). This explains why our underpredicted example molecules of MA=9 (Supplementary Fig. 21b) and MA=14 (Supplementary Fig. 21d) were unusually underpredicted in our XGBoost model, as our model could only use their single peaks for predictive information, instead of the typical >30 and >50 peaks, respectively, for molecules with similar MA scores.

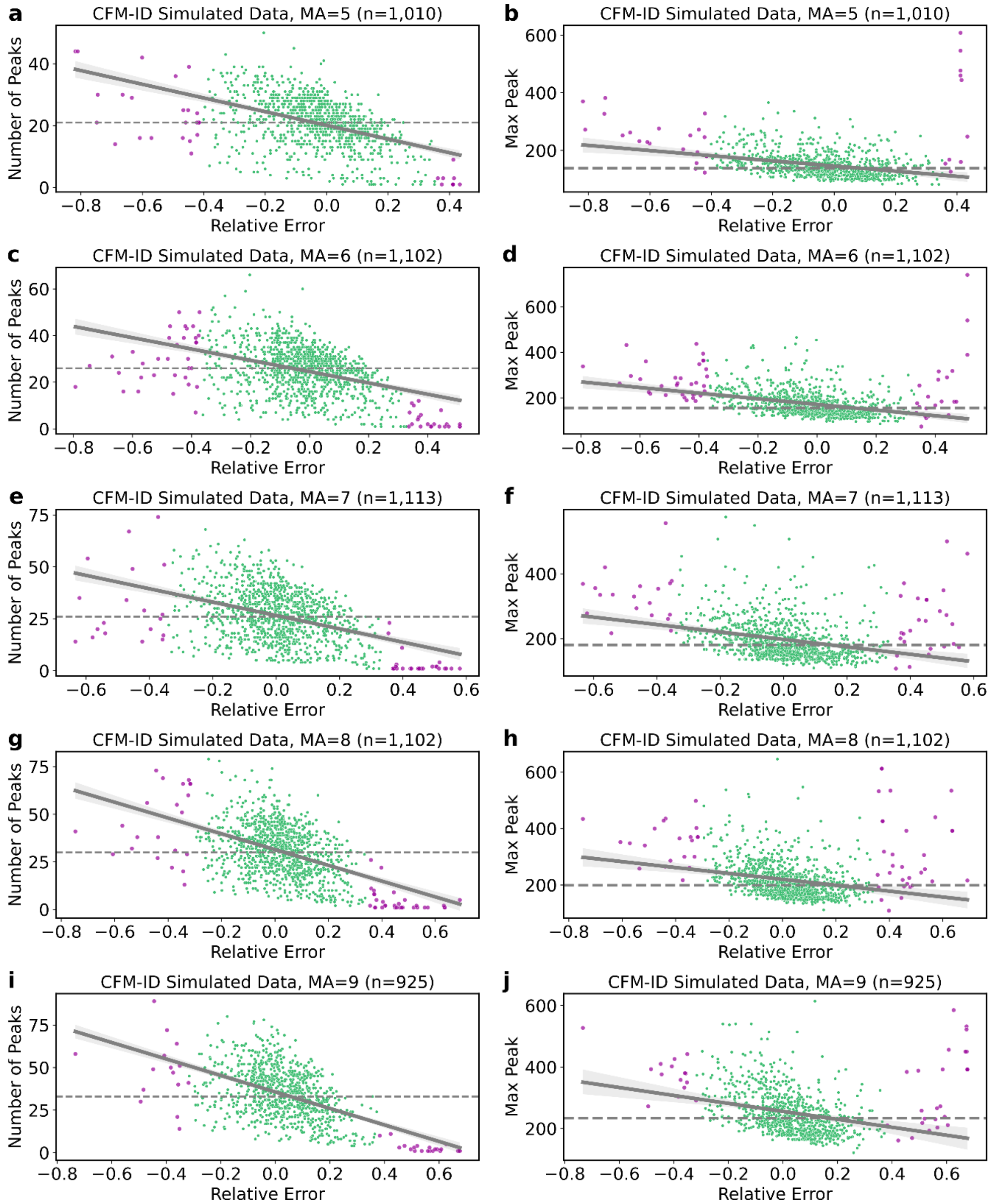

Supplementary Fig. 19: Relationship between MS peaks and error in XGBoost model. Relationship between peak information and relative error (calculated as $(y-\hat{y})/y$) for CFM-ID simulated 40eV MS for molecules with MA scores from 5 to 9 (from top to bottom plots). **a, c, e, g, i,** Number of peaks and relative error. **b, d, f, h, j,** Maximum peak and relative error. Outliers (defined as <Q1−IQR×1.5 or >Q3+IQR×1.5, where IQR is the interquartile range) are represented in pink, the median value of the peak information is represented as a horizontal dotted grey line, and the best fit with 95% confidence interval is represented as the diagonal grey line. In general, outlier molecules with overprediction (negative relative error) have many peaks and often a high maximum peak, and outlier molecules with underprediction (positive relative error) have a small number of peaks and often a high maximum peak.

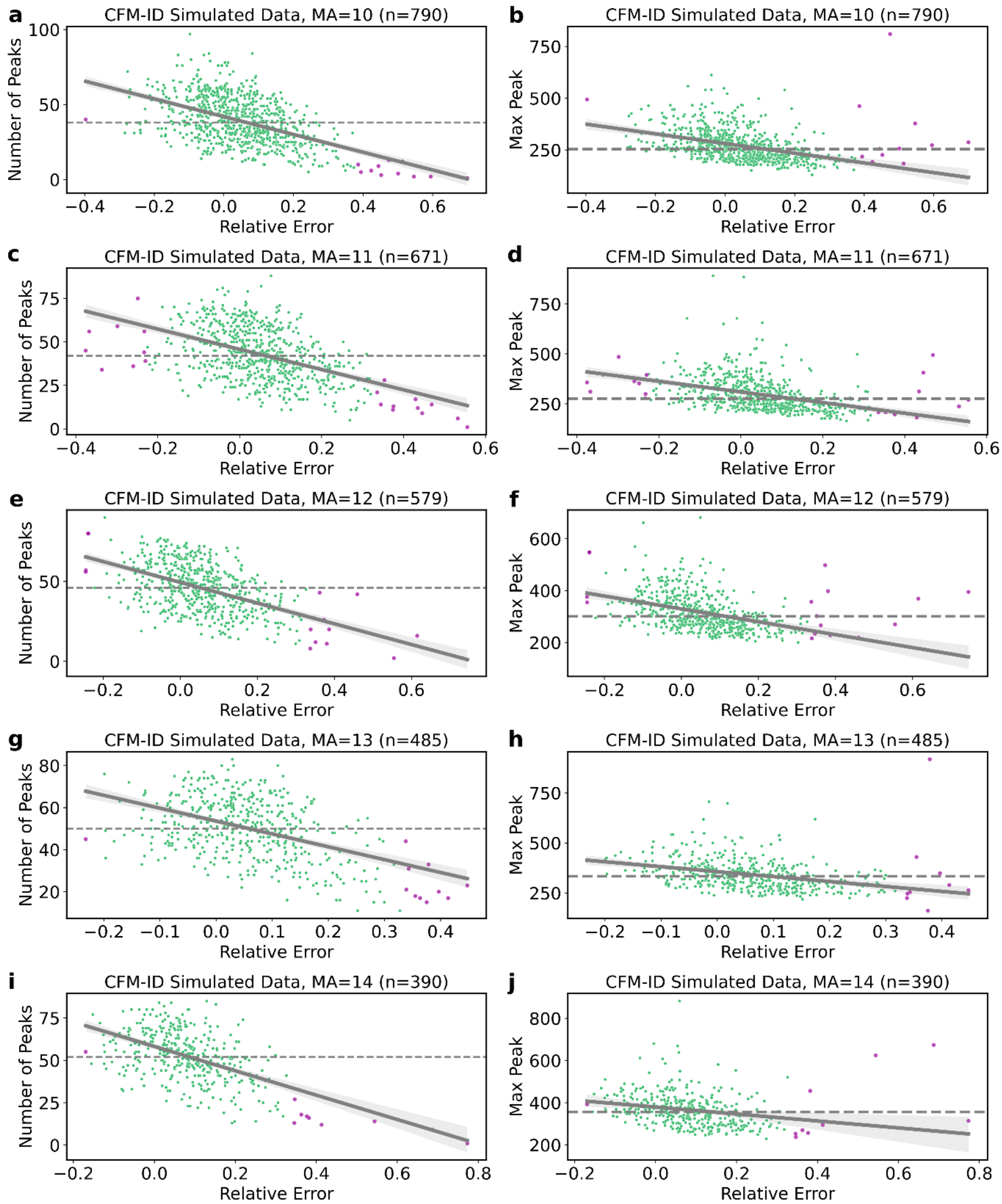

Supplementary Fig. 20: Relationship between MS peaks and error in XGBoost model. Relationship between peak information and relative error (calculated as $(y - \hat{y})/y$) for CFM-ID simulated 40eV MS for molecules with MA scores from 10 to 14 (from top to bottom plots). **a, c, e, g, i,** Number of peaks and relative error. **b, d, f, h, j,** Maximum peak and relative error. Outliers (defined as <Q1−IQR×1.5 or >Q3+IQR×1.5, where IQR is the interquartile range) are represented in pink, the median value of the peak information is represented as a horizontal dotted grey line, and the best fit with 95% confidence interval is represented as the diagonal grey line. In general, outlier molecules with overprediction (negative relative error) have many peaks and often a high maximum peak, and outlier molecules with underprediction (positive relative error) have a small number of peaks and often a high maximum peak.

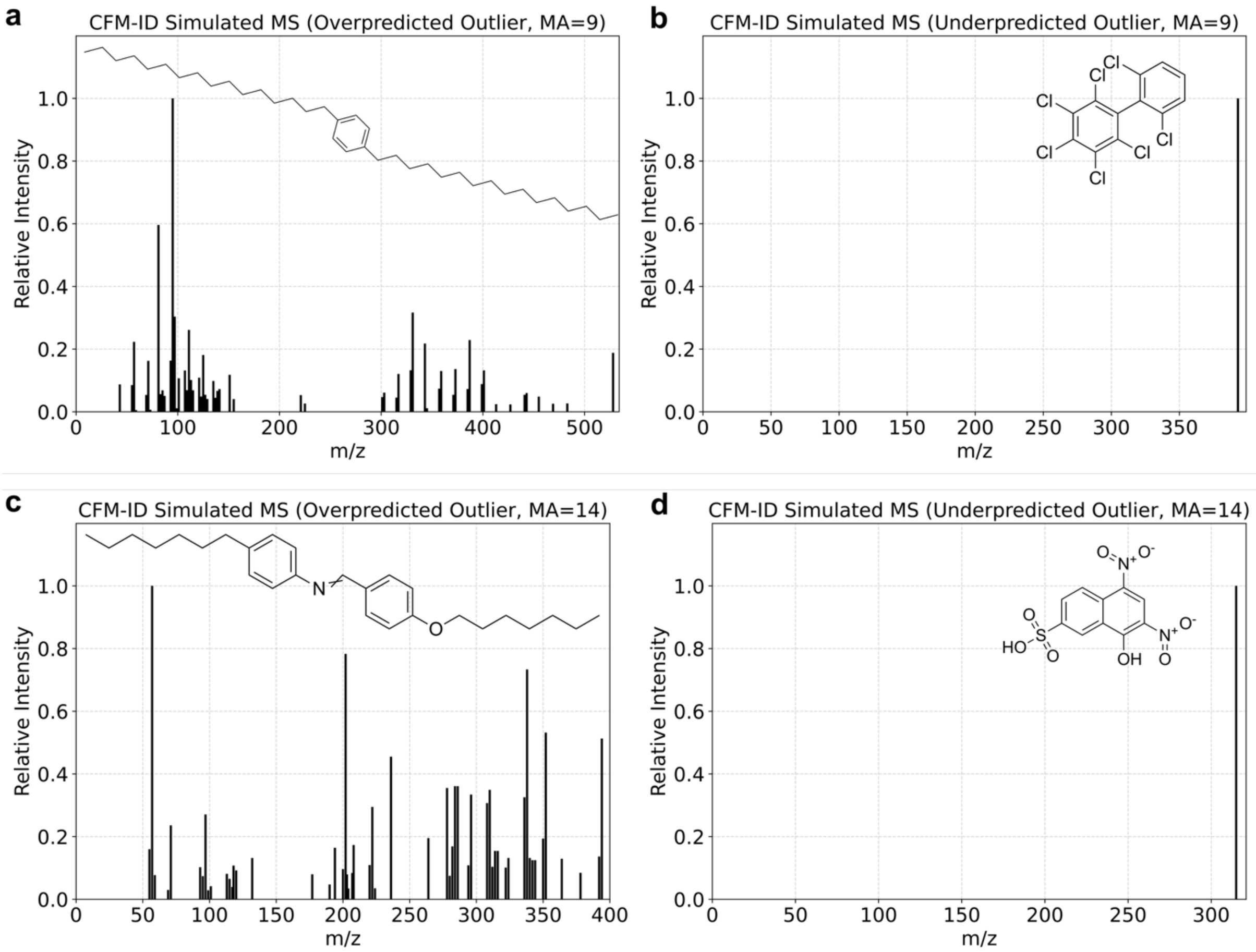

Supplementary Fig. 21: Example molecules with large error in XGBoost model. a, b, Example molecules with MA=9 for an overprediction case (**a**) and an underprediction case (**b**). The overpredicted molecule (i.e. XGBoost overestimated its MA score) contained long hydrocarbon chains (that are easy to fragment) and produced many peaks, while the underpredicted molecule (i.e. XGBoost underestimated its MA score) contained benzene rings with surrounding halogens that cause steric hindrance (making them harder to fragment) and produced only one peak. **c, d,** Example molecules with MA=14 for an overprediction case (**c**) and an underprediction case (**d**). The overpredicted molecule again contained long hydrocarbon chains (that are easy to fragment) and produced many peaks, while the underpredicted molecule contained fused benzene rings (that are hard to fragment) and produced only one peak.

This post-hoc analysis adds to evidence that our XGBoost model itself works well with predicting MA scores from MS data, as seen earlier with our smooth and convergent learning curve (Supplementary Fig. 14). The current limitations are not so much due to the model itself, but due to MS instrumentation constraints. It seems that multimodal approaches could indeed improve our model even more. Just as our integrated-energy model outperformed each of our single-energy models, a multimodal model that incorporated a wider range of collision energy

levels (with some even harsher than 40eV) and a range of ionization methods (EI and ESI) could possibly allow for fragmentation of molecules that are usually too stubborn to break. It is likely our model would effectively learn from multi-modal information and provide even better predictions. This is indeed an avenue for future work to expand upon in our attempt to infer molecular complexity as a biosignature during upcoming astrobiology missions.

5. Identifiers of example molecules

The SMILES identifiers for the 6 molecules shown in Fig. 2 are shown below.

'c1ccc2cc3cc4c(cc3cc2c1)[nH]c1cc2ccccc2cc14'

'C1CCCCCCCCCCCCCCCCCCCCCCCCCCCC1'

'C[Si](C)(C)OC[C@@H](O[Si](C)(C)C)[C@@H](O[Si](C)(C)C)[C@@H](O[Si](C)(C)C)C(=O)O[Si](C)(C)C'

'C12C3C4C5C1C1C6C2C2C3C3C4C4C5C1C1C6C2C3C41'

'CN1C(=O)C(O[Si](C)(C)C(C)(C)C)N=C(c2ccccc2)c2cc(Cl)ccc21'

'c1cc2ccc3ccc4ccc5ccc6ccc1c1c2c3c4c5c61'

The SMILES identifiers for the 3 molecules shown in Fig. 3 are shown below.

'C1=CC=C2C3=C(C=CC2=C1)C1=CC=CC2=C1C3=CC=C2'

'C[C@H]1CC[C@@]2(OC1)O[C@H]1C[C@H]3[C@@H]4CC[C@]5(O)C[C@@H](O[C@@H]6OC[C@H](O)[C@H](O)C6O)C[C@@H](O)[C@]5(C)[C@H]4CC[C@]3(C)[C@H]1[C@@H]2C'

'O=C(NCCN1CCOCC1)[C@H]1C[C@@H](O)[C@H](O)[C@@H]2N/C(=N\c3ccc(-n4cccn4)cc3)S[C@@H]21'

The SMILES identifiers for the 12 molecules used in the symmetry breakage study in Fig. 4 are shown below.

Dodecane:

'CCCCCCCCCCCC'

'CCCCCCCCCNCC'

'CCCCCCNCCNCC'

Cubane:

'C12C3C4C1C1C2C3C41'

'C12[C@H]3[C@H]4[C@H]1[C@H]1[C@@H]2[C@@H]3N41'

'[C@H]12[C@H]3[C@H]4[C@@H]5[C@H](N14)N2[C@H]35'

Coronene:

'c1cc2ccc3ccc4ccc5ccc6ccc1c1c2c3c4c5c61'

'C1=Cc2ccc3ccc4ccc5c6c4c3c2C2C1=CC=C(C=C5)N62'

'C1=Cc2ccc3ccc4ccc5c6c4c3c2C2C1=CC=C(N=C5)N62'

Saturated Buckminsterfullerene:

'C12C3C4C5C1C1C6C7C2C2C8C3C3C9C4C4C%10C5C5C1C1C6C6C%11C7C2C2C7C8C3C3C8C9C4C4C9C%10C5C5C1C1C6C6C%11C2C2C7C3C3C8C4C4C9C5C1C1C6C2C3C41'

'C12C3C4C5C1C1C6C7C8C1C1C5C5C9C4C4C%10C3C3C%11C2C6C2C6C7C7C%12C8C8C1C5N1C5C9C4C4C9C%10C3C3C(C%112)C2C6C7C6C7C%12C8C1C1C5C4[C@@H]4[C@H]9[C@H]3[C@H]2[C@H]6[C@@H]4C71'

'C12C3C4C5C6C1C1C7C2C2C8C3C3C4C4C9C5C5C6C6C1[C@@H]1[C@@H]%10C7C7C2C2C%11C%12C%13C%14C%15C%16C%17C%13C%11C7[C@@H]%10[C@H]%17[C@@H]7C%16C%10C(C6[C@H]17)C5N1C%10C%15C5C%14C6C%12C(C28)C3N6C4C5C91'

The SMILES for the 7 example molecules from Table 3, which showed improved MA predictions using XGBoost over baseline models, are provided below.

'CCCCCCCCCCCCCCCCCCCCCCCCCCCCCCCCCCCCCCCCCCCCCCCCCCCC'

'CCCCCCCC/C=C/CCCCCCCC(=O)OCC(COC(=O)CCCCCCC/C=C/CCCCCCCC)OC(=O)CCCCCCC/C=C/CCCCCCCC'

'Brc1cc(Br)c(-c2cc(Br)c(Br)cc2Br)cc1Br'

'C[Si](C)(C)O[C@H]1[C@H](O[Si](C)(C)C)[C@@H](O[Si](C)(C)C)[C@H](O[Si](C)(C)C)[C@@H](O[Si](C)(C)C)[C@H]1O[Si](C)(C)C'

'IC(I)=C(I)I'

'Cc1c(Br)c(Br)c(O)c(Br)c1Br'

'CC(C)CCCOC(=O)CN(C)C(=O)c1ccc(F)cc1'

The SMILES identifiers for the 3 molecules shown in Supplementary Fig. 6 are shown below.

'CCCCN1COCCOCCO1'

'C=C1C(=O)C[C@@H]2C(=C)[C@@H](O)C[C@H]3C(=C)C(=O)O[C@@H]3[C@@H]12
'

'OC1=CC(O)=C(OC2=CC(O)=C(O)C(OC3=CC(O)=C(O)C(O)=C3OC3=CC(O)=C(OC4=C
C(O)=C(O)C(O)=C4OC4=CC(O)=C(O)C(OC5=CC(O)=C(O)C(O)=C5)=C4)C(O)=C3)=C2)
C(O)=C1'

The SMILES identifiers for the 3 molecules shown in Supplementary Fig. 7 are shown below.

'O=C1OC(=O)C2=C1CC1=CC=CC=C1C2'

'O=C(O)CNC1=NC(=O)C2=NC=NC2=N1'

'OC[C@H]1O[C@H](O)[C@H](O)[C@@H](O)[C@@H]1O[C@@H]1O[C@H](CO[C@H
]2OC[C@@H](O[C@@H]3O[C@H](CO[C@H]4OC[C@@H](O[C@@H]5O[C@H](CO[
C@H]6OC[C@@H](O)[C@H](O)[C@H]6O)[C@@H](O)[C@H](O)[C@H]5O)[C@H](O)[
C@H]4O)[C@@H](O)[C@H](O)[C@H]3O)[C@H](O)[C@H]2O)[C@@H](O)[C@H](O)[
C@H]1O'

The SMILES identifiers for the 3 molecules shown in Supplementary Fig. 8 are shown below.

'C[C@H]1O[C@H](O[C@@H]2[C@H](O)[C@@H](O)[C@@H](O)O[C@@H]2C)[C@H]
(O)[C@@H](O)[C@H]1O'

'CC(C)=CC(=O)O[C@H]1[C@@H](O)[C@H](O)O[C@@H]([C@H]2C3=CC=CC(O)=C3
C(=O)C3=C(O)C=C(C)C=C32)[C@H]1O'

'CC(C)CCCCC=CCCCCCCCC(=O)ONCCCOC1=C(Br)C=C(CC(=NO)C(=O)NCCC2=CN
C(N)=N2)C=C1Br'

The SMILES identifiers for the 3 molecules shown in Supplementary Fig. 9 are shown below.

'[O-][S@@+]1C[C@H](O)[C@H](O)[C@@H](O)C[C@H](O)C[C@H](O)[C@@H](O)[C@H](O)[C@@H](O)C1'

'C[C@H]1CC[C@@]2(C(=O)O)CC[C@@]3(C)C(=CC[C@@H]4[C@@]5(C)CC(=O)[C@@H](O)C(C)(C)[C@@H]5CC[C@@]43C)[C@@H]2[C@@]1(C)O'

'COC1=C(C2=CC=CC=C2)C(O)=C(O)C(C2=CC=CC=C2)=C1C1=CC=C(O)C2=C1C=CC1=C2OC2=C(C3=CC=CC=C3)C(O)=C(O)C(C3=CC=CC=C3)=C21'

The SMILES identifiers for the 3 molecules shown in Supplementary Fig. 10 are shown below.

'C(C#CC1=CC=CC=C1)#CC1=CC=CC=C1'

'CC1=CC[C@H](O)[C@]2(C)CCC3=C(CO)C(=O)O[C@@H]3[C@@H]12'

'OC[C@@H]1O[C@@H](O[C@H]2[C@H](OC[C@@H]3O[C@H](O)[C@H](O)[C@H]3O)O[C@@H](CO)[C@@H]2O)[C@H](O)[C@H]1O'

The SMILES identifiers for the 4 example PubChem technosignature molecules shown in Supplementary Fig. 13 are provided below.

'O=P(O)(O)O[B-](O)(O)Cn1cc(CNS(=O)(=O)CC(F)(F)F)nn1'

'CC(C)(C)[Si](C)(C)OCC#Cc1nc(OS(=O)(=O)C(F)(F)F)cs1'

'CCC(C)SP(=O)(NS(=O)(=O)C(F)(F)F)N(C)C(=O)C(=O)Oc1ccc([N+](=O)[O-])cc1'

'COC(=O)C(=C(C)OS(=O)(=O)C(F)(F)F)N1C[C@H](SSc2cccc3sc([C@@H](C)O[Si](C)(C)C(C)(C)C)nc23)C1=O'

The SMILES identifiers for the 4 example outlier molecules shown in Supplementary Fig. 21 are provided below.

'CCCCCCCCCCCCCCCCc1ccc(CCCCCCCCCCCCCCCC)cc1'

'Clc1cccc(Cl)c1-c1c(Cl)c(Cl)c(Cl)c(Cl)c1Cl'

'CCCCCCCOc1ccc(C=Nc2ccc(CCCCCCC)cc2)cc1'

'O=[N+]([O-])c1cc([N+](=O)[O-])c2ccc(S(=O)(=O)O)cc2c1O'

6. Supplementary file references

1. Linstrom, P. NIST Chemistry WebBook, NIST Standard Reference Database 69. National Institute of Standards and Technology https://doi.org/10.18434/T4D303 (1997).
2. Horai, H. *et al.* MassBank: a public repository for sharing mass spectral data for life sciences. *J. Mass Spectrom.* **45**, 703–714 (2010).
3. MassBank consortium and its contributors. MassBank/MassBank-data: Release version 2025.05.1. Zenodo https://doi.org/10.5281/ZENODO.3378723 (2025).
4. Wang, F. *et al.* CFM-ID 4.0: More Accurate ESI-MS/MS Spectral Prediction and Compound Identification. *Anal. Chem.* **93**, 11692–11700 (2021).
5. Seet, I., Patarroyo, K. Y., Siebert, G., Walker, S. I. & Cronin, L. Rapid Exploration of the Assembly Chemical Space of Molecular Graphs. *J. Chem. Inf. Model.* acs.jcim.5c01964 (2025) doi:10.1021/acs.jcim.5c01964.
6. Böttcher, T. An Additive Definition of Molecular Complexity. *J. Chem. Inf. Model.* **56**, 462–470 (2016).
7. Greg Landrum *et al.* rdkit/rdkit: 2025_03_2 (Q1 2025) Release. Zenodo https://doi.org/10.5281/ZENODO.591637 (2025).

8. Bertz, S. H. The first general index of molecular complexity. *J. Am. Chem. Soc.* **103**, 3599–3601 (1981).

9. Pedregosa, F. *et al.* Scikit-learn: Machine learning in Python. *J. Mach. Learn. Res.* **12**, 2825–2830 (2011).

10. Chen, T. & Guestrin, C. XGBoost: A Scalable Tree Boosting System. in *Proceedings of the 22nd ACM SIGKDD International Conference on Knowledge Discovery and Data Mining* 785–794 (ACM, San Francisco California USA, 2016). doi:10.1145/2939672.2939785.

11. Cousins, K. R. ChemDraw Ultra 9.0. CambridgeSoft, 100 CambridgePark Drive, Cambridge, MA 02140. www. cambridgesoft. com. See Web site for pricing options. (2005).

12. Tosi, S. *Matplotlib for Python Developers*. (Packt Publishing Ltd, 2009).

13. Waskom, M. L. Seaborn: statistical data visualization. *J. Open Source Softw.* **6**, 3021 (2021).

14. Bah, T. *Inkscape: Guide to a Vector Drawing Program*. (prentice hall press, 2011).

15. Marshall, S. M. *et al.* Identifying molecules as biosignatures with assembly theory and mass spectrometry. *Nat. Commun.* **12**, 3033 (2021).

16. Chandrasekhar, V. *et al.* COCONUT 2.0: a comprehensive overhaul and curation of the collection of open natural products database. *Nucleic Acids Res.* **53**, D634–D643 (2025).

17. Becker, L., Bada, J. L., Winans, R. E. & Bunch, T. E. Fullerenes in Allende meteorite. *Nature* **372**, 507–507 (1994).

18. Kim, S. *et al.* PubChem 2025 update. *Nucleic Acids Res.* **53**, D1516–D1525 (2025).

19. Schmidt, G. A. & Frank, A. The Silurian hypothesis: would it be possible to detect an industrial civilization in the geological record? *Int. J. Astrobiol.* **18**, 142–150 (2019).

20. Lin, H. W., Abad, G. G. & Loeb, A. DETECTING INDUSTRIAL POLLUTION IN THE ATMOSPHERES OF EARTH-LIKE EXOPLANETS. *Astrophys. J.* **792**, L7 (2014).

21. Nair, A. S. *et al.* FDA-Approved Trifluoromethyl Group-Containing Drugs: A Review of 20 Years. *Processes* **10**, 2054 (2022).

22. Yale, H. L. The trifluoromethyl group in medical chemistry. *J. Med. Chem.* **1**, 121–133 (1958).

23. Fyfe, J. W. B. & Watson, A. J. B. Recent Developments in Organoboron Chemistry: Old Dogs, New Tricks. *Chem* **3**, 31–55 (2017).
